# Understanding Dietary Intervention-Mediated Epigenetic Modifications in Metabolic Diseases

**DOI:** 10.3389/fgene.2020.590369

**Published:** 2020-10-15

**Authors:** Shaza Asif, Nadya M. Morrow, Erin E. Mulvihill, Kyoung-Han Kim

**Affiliations:** ^1^University of Ottawa Heart Institute, Ottawa, ON, Canada; ^2^Department of Cellular and Molecular Medicine, Faculty of Medicine, University of Ottawa, Ottawa, ON, Canada; ^3^Department of Biochemistry, Microbiology and Immunology, Faculty of Medicine, University of Ottawa, Ottawa, ON, Canada

**Keywords:** non-alcoholic fatty liver disease, type 2 diabetes, obesity, dietary interventions, intermittent fasting, caloric restriction, DNA methylation, histone modification

## Abstract

The global prevalence of metabolic disorders, such as obesity, diabetes and fatty liver disease, is dramatically increasing. Both genetic and environmental factors are well-known contributors to the development of these diseases and therefore, the study of epigenetics can provide additional mechanistic insight. Dietary interventions, including caloric restriction, intermittent fasting or time-restricted feeding, have shown promising improvements in patients’ overall metabolic profiles (i.e., reduced body weight, improved glucose homeostasis), and an increasing number of studies have associated these beneficial effects with epigenetic alterations. In this article, we review epigenetic changes involved in both metabolic diseases and dietary interventions in primary metabolic tissues (i.e., adipose, liver, and pancreas) in hopes of elucidating potential biomarkers and therapeutic targets for disease prevention and treatment.

## Introduction

The continuous rise in metabolic diseases, such as obesity, type 2 diabetes (T2D; Table 1) and non-alcoholic fatty liver disease (NAFLD), is one of the leading causes of patient morbidity and mortality worldwide (Saklayen, 2018) and cannot be solely explained by the contribution of genetic and environmental factors. Indeed, epigenetics, which constitutes the reversible and heritable change in gene expression without modification of the underlying nucleotide sequence, serves as a mechanistic bridge. Epigenetic changes influenced by environmental cues can result in altered gene expression associated with metabolic function and dysfunction.

**TABLE 1:** Description of commonly used acronyms.

**Acronym**	**Description**
AcAc	Acetoacetate
ADF/EODF	Alternate day fasting or Every-other-day fasting
BAT	Brown adipose tissue
BHB	β-hydroxybutyrate
BMI	Body mass index
CR	Caloric restriction
DNL	*de novo* lipogenesis
DNMT	DNA methyltransferase
FA	Fatty acid
FAO	Fatty acid oxidation
FMD	Fasting-mimicking diet
GSIS	Glucose-stimulated insulin secretion
GWAS	Genome-wide association study
HAT	Histone acetyltransferase
HCC	Hepatocellular carcinoma
HDAC	Histone deacetylase
HDM	Histone demethylase
HFD	High-fat diet
HMT	Histone methyltransferase
HSC	Hepatic stellate cell
IF	Intermittent fasting
KD	Ketogenic diet
NAFLD	Non-alcoholic fatty liver disease
NASH	Non-alcoholic steatohepatitis
ROS	Reactive oxygen species
SCFA	Short-chain fatty acid
SNP	Single-nucleotide polymorphism
T2D	Type 2 diabetes
TG	Triglyceride
TRF	Time-restricted feeding
VLDL	Very-low-density lipoprotein
WAT	White adipose tissue

Overnutrition, especially of highly processed foods (Hall et al., 2019), accompanied by erratic diurnal eating patterns, constitute the major environmental contributors to the epidemic state of metabolic diseases today. As such, switching to a regular, nutritious diet can promote processes of maturation and restoration, and protect against the development of chronic metabolic disorders (Di Francesco et al., 2018). Since the applicability of pharmacological interventions in the treatment of metabolic disorders is limited by issues regarding off-target effects, patient compliance and tolerability, as well as lack of sufficiency in disease management (Longo and Panda, 2016); dietary interventions have become a promising, low-risk alternative or supplementary form of therapy. By adjusting meal timing and/or content, dietary interventions have shown continued success in reducing risk factors, inducing beneficial pleiotropic effects and ameliorating disease states (Longo and Panda, 2016).

**FIGURE 1:**
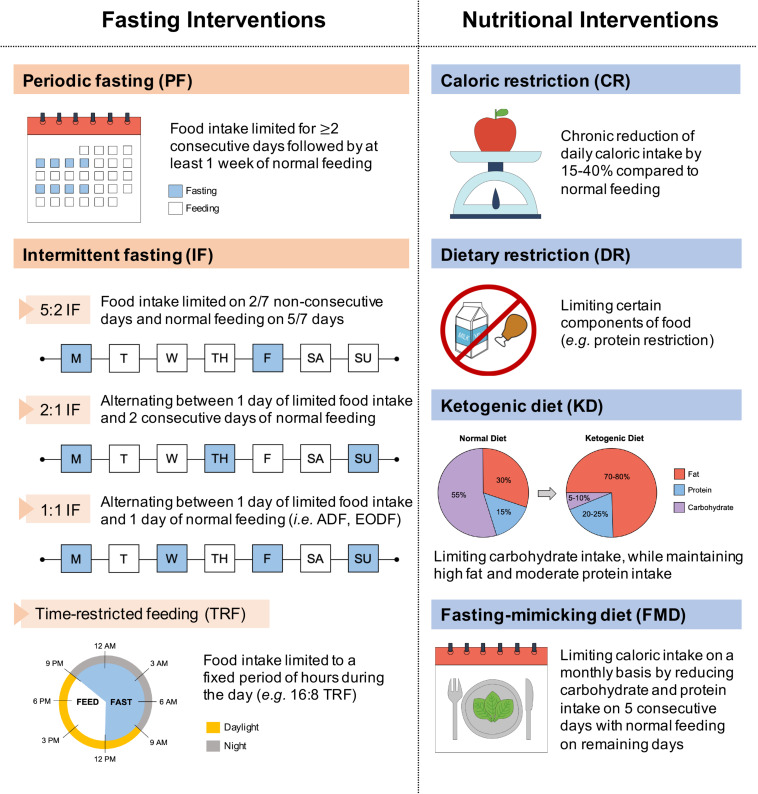
Classification of dietary interventions. Dietary interventions can be broadly categorized according to varied meal timing (fasting interventions) and meal content (nutritional interventions). Fasting interventions can be further subdivided into periodic fasting (PF) on a monthly basis and intermittent fasting (IF) on a weekly (5:2, 2:1, 1:1 IF) or daily (TRF) basis. ADF, alternate-day fasting; EODF, every-other-day fasting.

These dietary interventions involve limiting food intake of entire (i.e., fasting interventions) or selected nutrient compositions (i.e., nutritional interventions), without disturbing energy balance or inducing malnutrition. Specifically, fasting interventions can be categorized into intermittent fasting (IF) and periodic fasting (PF), where food intake is limited either on a daily/weekly basis or on a monthly basis, respectively (Anton et al., 2018; Yong-Quan Ng et al., 2019; Figure 1). IF cycles typically last 24 h and are separated by one or more days, whereas PF cycles last two or more days and are separated by at least a week (Longo and Mattson, 2014). Different forms of IF vary in their timing of meals and include the daily time-restricted feeding (TRF), and the weekly 5:2, 2:1, or 1:1 IF regimens. Moreover, nutritional interventions vary in their meal content and include caloric restriction (CR), dietary restriction (DR), ketogenic diet (KD), and fasting-mimicking diet (FMD).

Dietary interventions, such as CR and IF, extend lifespan and healthspan in various animal models, including yeast (Lin et al., 2000, 2002; Wu et al., 2013), worms (Wei et al., 2008; Honjoh et al., 2009; Uno et al., 2013), fruit flies (Grandison et al., 2009; Catterson et al., 2018; Villanueva et al., 2019), rodents (Goodrick et al., 1982; Hatori et al., 2012; Chaix et al., 2014; Rusli et al., 2017; Mitchell et al., 2019), and monkeys (Bodkin et al., 2003; Colman et al., 2009; Mattison et al., 2017). Studies done in humans also demonstrate beneficial effects of dietary intervention, specifically regarding overall metabolic improvements in body weight and fat mass (Heilbronn et al., 2005; Johnson et al., 2007; Varady et al., 2009; Harvie et al., 2011; Klempel et al., 2013; Varady et al., 2013; Redman et al., 2018; Anton et al., 2019; Ravussin et al., 2019; Stekovic et al., 2019; Wilkinson et al., 2020), circulating triglyceride (TG) and cholesterol levels (Johnson et al., 2007; Varady et al., 2009; Harvie et al., 2011; Klempel et al., 2013; Varady et al., 2013; Stekovic et al., 2019; Wilkinson et al., 2020), insulin sensitivity and glucose homeostasis (Halberg et al., 2005; Harvie et al., 2011; Sutton et al., 2018; Jamshed et al., 2019), and oxidative stress and inflammation (Johnson et al., 2007; Meydani et al., 2011; Redman et al., 2018; Sutton et al., 2018; Stekovic et al., 2019). Notably, the metabolic benefits of dietary interventions are not completely dependent on total caloric intake. For instance, the 2:1 IF regimen in mice provides comparable metabolic outcomes against obesity and associated metabolic dysfunctions, despite no difference in caloric intake (i.e., isocaloric) in comparison to *ad libitum* (i.e., normal feeding) (Kim K.H. et al., 2017; Kim R.Y. et al., 2019; Kim Y.H. et al., 2019).

**FIGURE 2:**
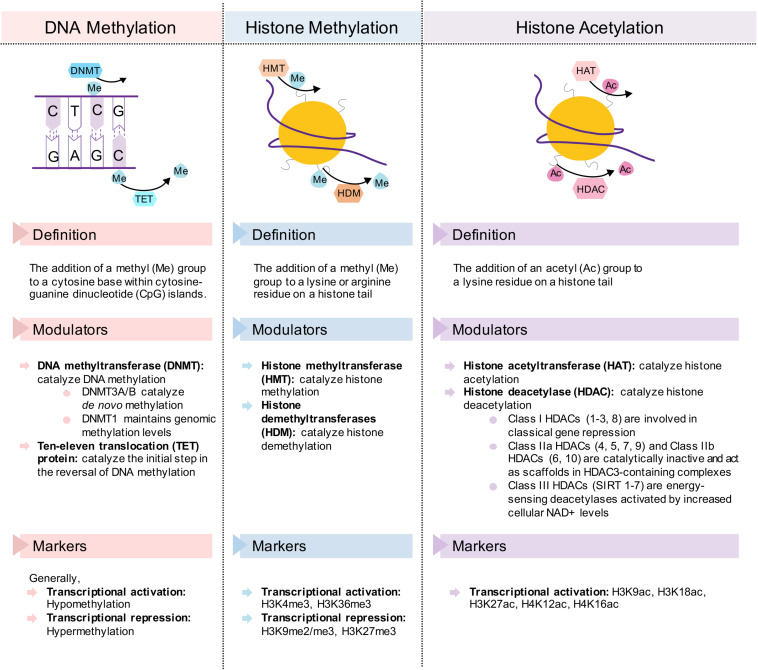
Description of the epigenetic change and its transcriptional modulators and markers in DNA methylation, histone methylation, and histone acetylation. Simplified diagrams show the forward and reverse reactions to each epigenetic mechanism.

These benefits conferred by dietary interventions involve cellular adaptations within various metabolic tissues, which are mediated by epigenetic modifications. Due to the plasticity of epigenetic factors, environmental changes, such as dietary interventions, which alter food intake and composition, have a significant impact on the epigenome. In this article, we will first review epigenetic changes in metabolic disease with a particular emphasis on adipose tissues, liver, and pancreas. We will primarily focus on DNA methylation and post-translational histone modifications (Figure 2), with the exception of non-coding RNAs reviewed elsewhere (Deiuliis, 2016; Green et al., 2017). Next, we will discuss how fasting as a component of most dietary interventions and caloric restriction modulate epigenetic regulation in these tissues. To conclude, we will also briefly review the epigenetics of gut microbiota and ketone body metabolism in the context of dietary interventions. Overall, the understanding of both metabolic diseases and dietary interventions from an epigenetic perspective will provide new insights for metabolic disease prevention, management and treatment.

## Epigenetic regulation in adipose tissues during metabolic disease and dietary intervention

### Adipose Tissue in Health and Metabolic Disease

Obesity is a serious metabolic disease that has reached worldwide rates of over 27.5% for adults and 47.1% for children (Ng et al., 2014). It has been estimated that obesity is 40−70% inheritable, where genome-wide association studies (GWAS) account for 20% of the variation (Locke et al., 2015). As such, it is becoming increasingly clear that epigenetic modifications serve as a link between environmental and genetic causes of obesity.

Uncontrolled adipose tissue expansion and accompanying dysfunction drive obesity and associated metabolic pathogenesis (Choe et al., 2016). Adipocytes initially expand in size (i.e., hypertrophy) to accommodate increases in energy intake relative to energy expenditure. When adipocytes become lipid-engorged and can no longer store the excess energy, adipogenesis, the process by which pre-adipocytes differentiate into mature adipocytes, expands adipocyte number (i.e., hyperplasia) (Choe et al., 2016; Chait and den Hartigh, 2020).

**FIGURE 3:**
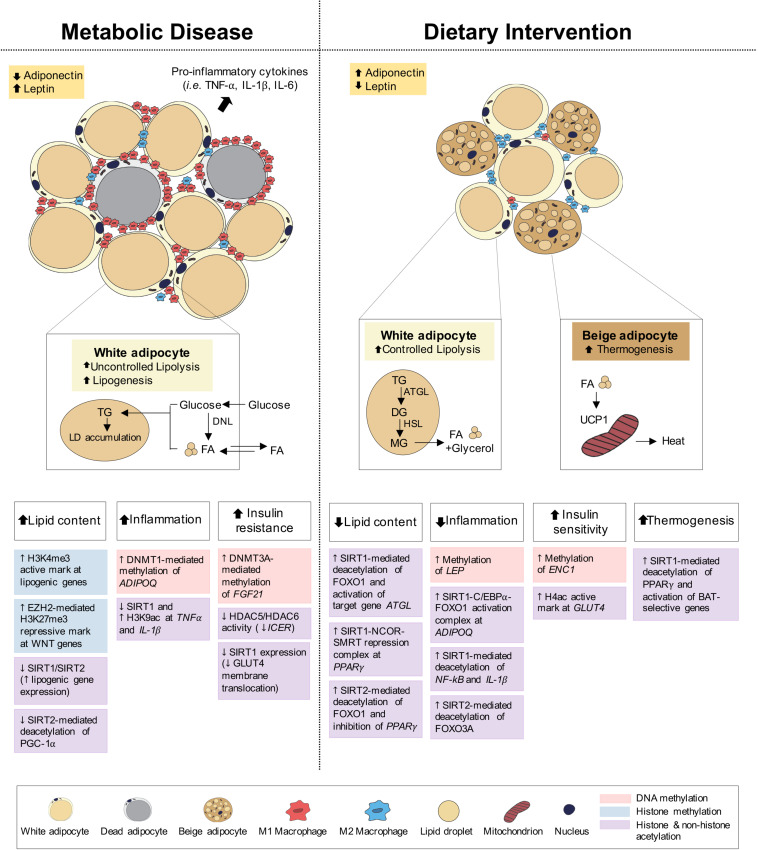
Epigenetic changes of adipose tissue in metabolic disease and dietary intervention. Adipose tissue in metabolic disease (i.e., obesity) predominantly consists of white adipocytes and presents with increased lipid content, inflammation and insulin resistance. Adipose tissue in dietary intervention is interspersed with both white and beige adipocytes and presents with reduced lipid content and inflammation as well as increased insulin sensitivity and thermogenesis. These physiological differences in adipose can be explained by epigenetic changes involving DNA methylation, histone methylation and histone and non-histone acetylation.

Adipose tissue is classified into white adipose tissue (WAT) and brown adipose tissue (BAT). WAT stores excess energy in the form of TG and is localized to subcutaneous (i.e., “beneath the skin”) and visceral depots (i.e., “surrounding internal organs”). BAT, by contrast, utilizes stored energy to produce heat in response to stimuli like cold stress, primarily via the uncoupling protein-1 (UCP-1), in a process known as non-shivering thermogenesis (Figure 3). BAT is also distributed subcutaneously (e.g., under the clavicles and in the interscapular region) and viscerally (e.g., perivascular, periviscus and around solid organs) (Sacks and Symonds, 2013; Jung et al., 2019); however, the initial distribution and size of BAT, as found in infants and young children, decreases with age. Moreover, as the “whitening” of brown adipocytes via dysregulated adipogenesis is associated with the development of obesity (Shimizu et al., 2014; Pellegrinelli et al., 2016; Longo et al., 2019), the “browning” of white adipocytes leading to increased thermogenesis may have therapeutic potential in the treatment of obesity (Choe et al., 2016).

Under feeding conditions, adipocytes take up and store circulating glucose and fatty acids (FA) through processes mediated by insulin-stimulated glucose transporter type 4 (GLUT4) translocation to the adipocyte cell surface and lipoprotein lipase (LPL) activity, respectively. Under fasting conditions, adipocyte lipolysis leads to FA release into circulation for use in other metabolic tissues, including muscle, kidney, gut and liver (Ahima and Flier, 2000; Choe et al., 2016; Figure 3). Adipose tissues also function as endocrine organs through secretion of cytokines (e.g., TNFα, IL-1, and IL-6) and hormones (e.g., leptin, adiponectin), to mediate pathways of energy homeostasis, adipocyte differentiation, insulin sensitivity and inflammatory control (Wozniak et al., 2009). The reduction in insulin receptor density within the expanded adipose tissue and the subsequent development of insulin resistance both promote the uncontrollable release of FA, leading to aberrant lipid accumulation and lipotoxicity in peripheral tissues (Choe et al., 2016; Muir et al., 2016; Longo et al., 2019). Lipotoxicity, in combination with reduced anti-inflammatory adiponectin and increased pro-inflammatory cytokine secretion, promotes systemic inflammation associated with obesity, T2D and NAFLD.

### Epigenetic Changes in Adipose Tissues in Metabolic Disease

#### DNA Methylation/Demethylation

DNA methylation is a key epigenetic modification involved in adipose development and function. Changes in DNA methylation in adipocytes have been associated with both the cause and effect of metabolic dysregulation in obesity, where hypomethylation appears to be the dominating change (Sonne et al., 2017). A genome-wide screen has identified 625 significant differentially methylated regions (DMRs) associated with diet-induced obesity phenotypes, of which 232 DMRs correlate with high-fat diet (HFD) alone, and 249 regions are conserved in adipose tissue from obese subjects. Among these, 30 single-nucleotide polymorphisms (SNPs) are associated with T2D (Multhaup et al., 2015). As shown in Table 2, increased and decreased DNA methylations generally correspond to genes involved in positively (i.e., glucose homeostasis) and negatively (i.e., inflammation) regulating adipocyte metabolism, respectively. Additionally, DNA methylation profiles of diet-induced and genetically obese (i.e., *ob/ob*) mice revealed that methylation changes are more abundant in visceral than subcutaneous adipocytes (Sonne et al., 2017), with visceral fat being the greater contributor to obesity and its associated metabolic dysfunctions (Fox et al., 2007; Neeland et al., 2013).

**TABLE 2:** Changes in DNA methylation and gene expression in adipose tissue associated with obesity and high-fat feeding.

**Gene**	**Function in adipose tissue**	**Direction of methylation change**	**Change in gene expression**	**References**
*Aacs*	Fatty acid and cholesterol synthesis	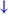	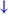	Sonne et al., 2017
*Abcd2*	Fatty acid transport	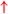	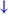	Multhaup et al., 2015
*Adipoq*	Glucose utilization and insulin sensitivity	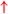	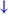	Kim et al., 2015
*ADRBK1*	Insulin and G protein-coupled receptor signalling	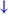	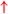	Multhaup et al., 2015
*Akt2*	Insulin-mediated glucose uptake	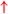	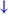	Multhaup et al., 2015
*Ankrd26*	Feeding behaviour and obesity development	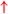	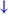	Raciti et al., 2017
*As3mt*	Methyltransferase, arsenic metabolism	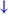	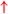	Multhaup et al., 2015
*Ehd2*	Regulation of adipocyte function	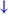	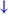	Sonne et al., 2017
*Fbxw8*	Development	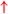	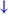	Multhaup et al., 2015
*FGF21*	Glucose uptake	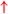	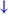	You et al., 2017
*FOXO1*	Adipocyte differentiation	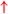	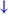	Multhaup et al., 2015
*Hoxa5*	Adipocyte differentiation	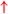	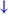	Parrillo et al., 2016
*Irf8*	Inflammation	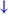	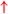	Sonne et al., 2017
*Kctd16*	Obesity risk	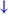	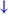	Sonne et al., 2017
*Lipe*	Lipolysis	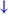	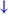	Sonne et al., 2017
*Mkl1*	Negative regulator of WAT browning	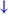	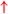	Multhaup et al., 2015
*Nrp2*	Lymphatic vessel development	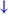	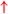	Sonne et al., 2017
*Pck1*	Gluconeogenesis	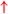	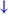	Multhaup et al., 2015
*Plekho1*	Glucose uptake	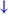	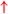	Multhaup et al., 2015
*Prcp*	Regulation of body weight	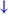	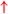	Sonne et al., 2017
*Reep6*	ER trafficking	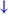	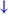	Sonne et al., 2017
*Rgs3*	G protein signaling	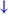	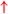	Multhaup et al., 2015
*Setd6*	Methyltransferase, inflammation	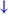	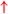	Sonne et al., 2017
*Shank3*	Synaptic function	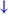	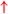	Sonne et al., 2017
*Sorbs1*	Insulin signaling	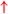	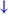	Multhaup et al., 2015
*Tcf7l2*	WNT signaling	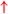	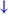	Multhaup et al., 2015
*Tnfaip8l2*	Glucose uptake, inflammation	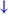	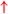	Multhaup et al., 2015
*Vps13c*	Adipocyte differentiation	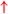	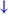	Multhaup et al., 2015

*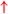 = indicates increased DNA methylation and/or gene expression; 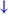 = indicates decreased DNA methylation and/or gene expression.*

##### Regulators of DNA methylation

DNA methyltransferases (DNMTs) catalyze DNA methylation. In contrast, ten-eleven translocation (TET) proteins catalyze the initial step of the reverse reaction (Figure 2; Jin and Robertson, 2013; Yong-Quan Ng et al., 2019). Altered expression of these DNA methylation modulators in adipose tissue can cause metabolic disease development and/or progression. High *DNMT1* expression is found in adipocytes of obese humans (Kim et al., 2015). Similarly, increased *Dnmt1* mRNA level is found in WAT of HFD-fed and genetically obese (i.e., *db/db*) mice compared to chow-fed and wild-type lean mice (Kim et al., 2015). *Dnmt1* expression and activity in mouse 3T3-L1 adipocytes are induced by the pro-inflammatory cytokines TNFα and IL-1β (Kim et al., 2015). A known target of DNMT1 is *Adipoq*, which encodes the key anti-diabetic and anti-inflammatory adipokine, adiponectin (Stern et al., 2016). Over-expression of *Dnmt1* in 3T3-L1 adipocytes increases methylation and decreases expression of *Adipoq*, while its knockdown results in the reverse (Kim et al., 2015), suggesting that direct hypermethylation and heterochromatin formation by DNMT1 at the *Adipoq* gene promoter is involved in obesity pathogenesis. In addition, the expression of *Dnmt3a*, not *Dnmt3b*, is increased in WAT of both diet- and genetically induced obese (i.e., *ob/ob*) mice (Parrillo et al., 2016; You et al., 2017) and the adipose-specific deletion of *Dnmt3a* in HFD-fed mice improves insulin sensitivity and glucose tolerance, independent of adiposity (You et al., 2017). Using an unbiased screen, the authors have identified *Fgf21* as a key target gene of DNMT3A. Correspondingly, adipose-specific *Dnmt3a* deletion leads to a decrease in the methylation and an increase in the expression of adipose *Fgf21* in both WAT and BAT (You et al., 2017). However, experiments using adipose tissue-specific *Dnmt3a* and *Fgf21* double knockout mice are still required to determine the mechanism of enhanced insulin sensitivity. Additionally, higher DNA methylation levels of *FGF21* have been observed in the WAT of T2D patients, which negatively correlated to *FGF21* mRNA expression, although DNMT3A levels were not measured (You et al., 2017).

#### Histone Methylation/Demethylation

Histone methylation, mediated by histone methyltransferases (HMTs) and reversed by histone demethylases (HDMs) (Figure 2), regulates adipogenesis through the addition and removal of activating and repressing histone methylation marks in adipocytes. Dysregulated adipogenesis through histone methylation impairs adipose tissue development and function and is associated with the maladaptive obesogenic condition.

##### H3K4 methylation (MLL3/MLL4, LSD1)

In adipose tissue of morbidly obese pre-diabetic patients, trimethylation of histone H3 at lysine 4 (H3K4me3), a gene activation mark, is found to be enriched at the promoters of genes associated with adipogenesis and lipid metabolism (e.g., *LPL*, *SREBF2*, *SCD1*, *PPARG*) (Castellano-Castillo et al., 2019), as well as at the E2F transcription factor 1 (*E2F1*), a contributor of obesity pathogenesis (Fajas et al., 2002; Haim et al., 2015; Denechaud et al., 2017). This finding suggests that the maintenance of H3K4me3 at promoters of adipogenic genes by HMTs and HDMs can be implicated in the development of obesity. MLL3/MLL4 (KMT2C/KMT2D) are H3K4 mono- and di-methyltransferases that mediate H3K4me3 transcriptional activation of adipogenic genes (e.g., *Pparg, Cebpa*) in association with the pax transactivation domain interacting protein (PTIP) co-factor (Lee et al., 2008, 2013; Cho et al., 2009). Additionally, LSD1 (KDM1A) catalyzes H3K4 mono- and di-demethylation to activate BAT-selective genes and to repress WAT-selective genes, in association with either nuclear receptor factor 1 (NRF1) or PR domain containing 16 (PRDM16), respectively (Hino et al., 2012; Duteil et al., 2014, 2016; Sambeat et al., 2016; Zeng et al., 2016). LSD1 also promotes BAT thermogenesis by repressing the glucocorticoid-activating enzyme, *HSD11B1*, thereby preventing the accumulation of excess glucocorticoid in adipose tissue (Zeng et al., 2016). Increased levels and secretion of glucocorticoid in adipose tissues are associated with obesity, insulin resistance and dyslipidemia (Akalestou et al., 2020). Notably, both mice lacking MLL3/MLL4 co-factor PTIP in adipose tissues (*aP2-Cre; Paxip^*flox/flox*^*) and LSD1 in adipose tissues or BAT specifically (*Adipoq-Cre; Lsd1^*flox/flox*^, Ucp1-Cre; Lsd1^*flox/flox*^*) exhibit similar obesogenic phenotypes with increased body weight and fat mass as well as dysfunctional BAT, indicated by lipid accumulation and reduced mitochondrial fatty acid oxidation (FAO) (Duteil et al., 2016; Zeng et al., 2016). Altogether, these results suggest that the H3K4 HMT/HDM balance is necessary for maintaining adipocyte function.

##### H3K9 methylation (EHMT1/EHMT2, LSD1, JHDM2A)

Both G9a (EHMT2, euchromatic histone lysine N-methyltransferase 2) and EHMT1 are histone H3 lysine 9 (H3K9) di- and tri-methyltransferases involved in maintaining the H3K9me2/me3 repressive mark. G9a inhibits adipogenesis while promoting the activation of Wnt/β-catenin signaling (Wang L. et al., 2013; Zhang et al., 2014) involved in brown/beige adipocyte development (Chen and Wang, 2018), and Ehmt1 regulates BAT-selective gene programs for BAT development and function in association with Prdm16 (Ohno et al., 2013). *G9a* (*aP2-Cre; Ehmt2^*flox/flox*^*) and *Ehmt1* (*Adipoq-Cre; Ehmt1^*flox/flox*^*) knockout mice develop increased adiposity (Ohno et al., 2013; Wang L. et al., 2013), while adipose tissue-specific *Ehmt1* knockout mice also present with reduced BAT thermogenesis and insulin resistance (Ohno et al., 2013). Interestingly, patients with Kleefstra syndrome can have a *9q34* chromosomal deletion containing the *EHMT1* gene and display childhood obesity, thus suggesting a plausible role for EHMT1 in obesity development (Cormier-Daire et al., 2003; Willemsen et al., 2012). In contrast, both LSD1 in association with the zinc finger protein 516 (Zfp516) (Sambeat et al., 2016), and Jhdm2a/Jmjd1a (KDM3A) in association with the chromatin remodeling complex SWI/SNF (Tateishi et al., 2009; Abe et al., 2015, 2018) catalyze the demethylation of H3K9me2/me3 to mediate transcriptional activation of BAT-selective genes (e.g., *Ucp1*, *Pgc-1α*) and stimulate thermogenic function. Consequently, abrogation of *Lsd1* (*Ucp1-Cre; Lsd1^*flox/flox*^*) (Sambeat et al., 2016) or *Jhdm2a* (*Jhdm2a*^–/–^) (Inagaki et al., 2009; Tateishi et al., 2009) in mice results in increased body weight, fat accumulation, and impaired glucose homeostasis, as well as “whitening” of BAT and dysregulated fatty acid metabolism. Taken together, these results suggest an important role for these H3K9 HMTs and HDMs in obesity resistance.

##### H3K27 methylation (EZH2)

Trimethylation of histone H3 at lysine 27 (H3K27me3), a repressive mark, is increased in the WAT of HFD-fed mice and obese patients (Yi et al., 2016). H3K27me3 is mediated by the Polycomb-Repressive Complex 2 (PRC2), which contains the Enhancer of Zeste Homolog 2 (EZH2) as the enzymatic component, catalyzing di- and tri-methylation of H3K27. EZH2 epigenetically represses Wnt genes (e.g., *Wnt1*, *−6*, *−10a*, *−10b*) to inhibit Wnt/β-catenin signaling while simultaneously promoting adipogenesis through the upregulation of *Pparg* and *Cebpa* (Wang L. et al., 2010; Yi et al., 2016; Jang et al., 2017). GSK126-mediated inhibition of EZH2 in HFD-induced obese mice reduces fat accumulation, improves glucose homeostasis, and increases adipose thermogenesis (Wu et al., 2018).

##### H3K36 methylation (NSD2)

Dimethylation of histone H3 at lysine 36 (H3K36me2), an activation mark, is found to be protective against impaired adipose tissue function associated with obesity. The nuclear receptor binding SET domain protein 2 (NSD2) HMT mediates dimethylation at H3K36 for the activation of PPARγ-dependent gene programs, critical for mature brown and white adipocyte function. The depletion of the Nsd2-mediated H3K36me2 mark in adipocytes disrupts thermogenic function with “whitening” of BAT and increases insulin resistance of WAT (Zhuang et al., 2018).

#### Histone Acetylation/Deacetylation

##### Class I/II HDACs

Histone acetylation and deacetylation involve the addition and removal of acetyl groups to lysine residues on histone tails, and are mediated by histone acetyltransferases (HATs) and histone deacetylases (HDACs), respectively (Figure 2). Total HDAC activity is decreased in the adipose tissue of obese individuals and HFD-fed mice (Bricambert et al., 2016), and mutations in HDAC4, a class II HDAC, have been associated with obesity (Williams et al., 2010). This reduced HDAC activity is mainly attributed to decreased HDAC5 and HDAC6 (class II HDAC) levels in WAT, which are accompanied by a decrease in inducible cAMP early repressor (Icer) function and an increase in its target activating transcription factor 3 (*Atf3*), associated with insulin resistance (Bricambert et al., 2016). Additionally, HDAC5 can interact with the GLUT4 enhancer factor (GEF) in adipocytes for the repression of *Glut4* promoter activity (Sparling et al., 2008; Weems and Olson, 2011), suggesting a plausible mechanism by which *Glut4* expression and insulin-mediated glucose uptake are dysregulated in obesity and T2D. Moreover, the expression of class I HDACs, *HDAC1* and *HDAC3*, is reduced in the adipose tissue of obese female patients (Jannat Ali Pour et al., 2020), however, their role in adipose tissue is not yet well understood.

##### Class III HDAC (SIRT1)

Sirtuin 1 (SIRT1), the mammalian ortholog of the yeast silent information regulator 2 (Sir2) protein, is a nuclear NAD^+^-dependent class III HDAC that catalyzes the removal of acetyl groups from protein substrates. SIRT1 is often termed the “master metabolic regulator” due to its ability to modulate the expression of several key metabolic transcription factors and co-factors in response to environmental stimuli (Schug and Li, 2011; Li, 2013). SIRT1 gene and protein expression is significantly reduced in adipose tissue of HFD-fed mice (Yoshizaki et al., 2009, 2010) as well as in chronically obese patients in which it negatively correlates with their body mass index (BMI) (Costa Cdos et al., 2010; Gillum et al., 2011; Perrini et al., 2020). *SIRT1* knockdown in human adipose progenitor cells results in a significant increase in cellular lipid content with elevated expression of adipogenic genes (*PPARG2*, *SREBF1C*, *FASN*, *ADIPOQ*, *SLC2A4*) (Perrini et al., 2020). Moreover, HFD-fed *Sirt1* heterozygous mice (*Sirt1^+/–^*) (Xu et al., 2016), adipose-specific *Sirt1*-KO mice (*aP2-Cre; Sirt1^*flox*/flox^*), and obese patients with decreased adipose *SIRT1* expression (Gillum et al., 2011) all exhibit increases in proinflammatory cytokine levels (IL-1β, TNFα, IL-10) and macrophage infiltration (Gillum et al., 2011; Xu et al., 2016) in WAT. Increased inflammation upon *Sirt1* deficiency is associated with increased H3K9 acetylation of *TNFα* and *IL-1β* promoter sites (Vaquero et al., 2004; Yoshizaki et al., 2009; Gillum et al., 2011; Liu et al., 2011) and reduced adiponectin levels (Qiao and Shao, 2006; Gillum et al., 2011). In addition, the HFD-fed *Sirt1* heterozygous mice (*Sirt1^+/–^*) present with more severe insulin resistance, compared with wild-type mice (Xu et al., 2016), which may be mediated by reductions in adipose GLUT4 translocation and insulin-stimulated glucose transport (Yoshizaki et al., 2009). These HFD-fed *Sirt1^+/–^* mice also exhibit reduced BAT thermogenesis as well as BAT degeneration indicated by mitochondrial dysfunction and loss. Taken together, these studies suggest a protective role of adipose SIRT1 in maintaining lipid and glucose homeostasis and inflammatory control, which is otherwise abrogated in the development of obesity and T2D.

##### Class III HDAC (SIRT2)

Sirtuin 2 is another NAD^+^-dependent class III HDAC, which in contrast to the nuclear SIRT1, is primarily cytoplasmic, but can transiently shuttle to the nucleus for deacetylation of transcription factors (de Oliveira et al., 2012; Gomes et al., 2015). *SIRT2* expression is found to be decreased in the adipose tissue of HFD-fed mice and obese patients (Krishnan et al., 2012; Perrini et al., 2020) and negatively correlates with their BMI, similar to SIRT1 (Perrini et al., 2020). In obesity, adipose SIRT2 expression is suppressed by adipose hypoxia-induced cellular hypoxia-inducible factor 1-α (HIF1α), which prevents SIRT2-mediated post-translational deacetylation and activation of PGC-1α and its FAO transcriptional gene program (Zhang X. et al., 2010; Krishnan et al., 2012). Similarly, *SIRT2* knockdown in isolated human adipose stem cells promotes adipogenesis and lipid accumulation through the induction of *PPARG2*, *SREBF1C*, *FASN*, *ADIPOQ*, and *SLC2A4* gene expression (Perrini et al., 2020), whereas *SIRT2* over-expression inhibits this process. Therefore, hypoxia-induced reductions of SIRT2 in obesity may contribute to adipocyte dysregulation by limiting oxidative capacity and increasing lipid mass.

Whether these functions of SIRT2 in adipocytes are mediated by its HDAC activity or cytoplasmic role remains unclear. Previous studies have demonstrated that SIRT2 regulates adipocyte differentiation through direct modulation of FOXO1 acetylation (Jing et al., 2007; Wang and Tong, 2009). On the other hand, SIRT2 controls mitosis by modulating histone H4K16 acetylation (Vaquero et al., 2006) and since mitotic clonal expansion is critical for adipocyte differentiation (Tang and Lane, 2012), this suggests that SIRT2 may regulate adipogenesis through histone modifications. Indeed, it has recently been shown that SIRT6, another class III HDAC, controls mitotic clonal expansion during adipogenesis by repressing kinesin family member 5C (KIF5C) expression with deacetylation of H3K9ac and H3K56ac at its promoter (Chen et al., 2017). Since the loss of *Sirt6* blocks adipogenesis and *Sirt6* mutant mice are extremely lean and die early with numerous severe metabolic abnormalities (Xiao et al., 2010), these results emphasize that proper development of adipocytes is critical for maintaining metabolic balance. Altogether, the lack of SIRT1-, SIRT2- and SIRT6-dependent deacetylation and activation of specific adipose gene programs can contribute to the development of metabolic conditions, including obesity and T2D.

### Epigenetic Changes in Adipose Tissues in Dietary Intervention

Modulation of lipid compartmentalization and efficient utilization of excess energy in adipose tissues are critical targets for the treatment of obesity and related metabolic dysfunctions. Dietary interventions, including IF and CR, markedly reduce adipocyte size and depot weights in rodent models of obesity (Wheatley et al., 2011; Kim K.H. et al., 2017; Liu B. et al., 2019; Miyamoto et al., 2019), and confer improvements in adipose tissue inflammation and insulin sensitivity (Anson et al., 2003; Wheatley et al., 2011; Duncan et al., 2016; Gotthardt et al., 2016; Kim K.H. et al., 2017; Li et al., 2017; Kim Y.H. et al., 2019; Liu B. et al., 2019; Figure 3). Additionally, adipose thermogenesis via the induction of WAT “browning”(beige fat) and activation of BAT appear to be predominant pathways (Hatori et al., 2012; Hatting et al., 2017; Kim K.H. et al., 2017; Li et al., 2017), which elevate energy expenditure, mitochondrial biogenesis and energy dissipating capacity (Harms and Seale, 2013; Cypess et al., 2015). Here, we summarize evidence for epigenetic links between dietary interventions and resulting metabolic improvements.

#### DNA Methylation/Demethylation

Female obese patients subjected to bariatric surgery with significantly reduced body weight (∼27%) and food intake show reductions in global DNA methylation levels and differentially methylated genes associated with obesity and T2D in adipose tissues (Benton et al., 2015; Dahlman et al., 2015), thus providing context for weight loss and adipocyte reprogramming. These genes are associated with the regulation of body weight (*LEPR*, *FTO*), cholesterol homeostasis (*CETP*, *LCAT*), blood glucose (*IRS1, INSR*) (Benton et al., 2015), adipose tissue function (*mTOR*, *RPTOR*) (Macartney-Coxson et al., 2017), and epigenetics (*FOXP2*, *HDAC4*, *DNMT3B*) (Benton et al., 2015). Studies investigating DNA methylation changes in adipose tissue upon dietary interventions, however, are limited. In one study, obese women on a 6-month CR diet (1100−1800 kcal/day) who lost >3% of their body fat showed hypermethylation at three genomic loci in their subcutaneous adipose tissue. Genes at these *loci* were associated with lipid (e.g., *PLCL4*) and glucose (e.g., *ENC1*) homeostasis and epigenetic regulation (e.g., *PRDM8*) (Bouchard et al., 2010). In particular, the ectodermal-neural cortex gene 1 (*ENC1*), previously associated with obesity (Zhao et al., 2000; Gerlini et al., 2018), was both differentially methylated (increased) and expressed (decreased) after CR treatment (Bouchard et al., 2010). In another study, 36-h of fasting in young, healthy men increased DNA methylation at the promoter site of *LEP* in subcutaneous adipose tissue, leading to a 3-fold decrease in plasma leptin levels (Hjort et al., 2017). Additionally, rosiglitazone, a PPARγ agonist, and a plausible CR-mimetic, mediates TET2-dependent demethylation of promoter regions of PPARγ target genes, such as *ADIPOQ* and *FABP4*, and results in enhanced insulin-stimulated glucose uptake in 3T3-L1 adipocytes (Bian et al., 2018). Altogether, these CR-related DNA methylation changes in adipose tissue can potentially be used as biomarkers of improved adiposity.

#### Histone Methylation/Demethylation

Histone methylation changes in adipose tissue as a result of dietary interventions have not yet been studied. Thus, studies showing similar alleviation of the disease state can be used to suggest analogous epigenetic mechanisms. For example, histone demethylases LSD1 of H3K4 (Duteil et al., 2014), JMJD1A of H3K9 (Abe et al., 2018), and UTX (KDM6A) (Zha et al., 2015), and JMJD3 (KDM6B) (Pan et al., 2015) of H3K27 mediate the induction of BAT-selective genes (e.g., *Ucp1*, *Pgc-1α*, *Ppara*, *Cidea*) in WAT for the development of thermogenically active beige adipocytes. Consequently, whole-body *Lsd1* over-expressing mice (*Rosa26-Lsd1*) present with reduced body weight gain and increased energy expenditure, associated with smaller adipocyte size and greater mitochondrial content in WAT (Duteil et al., 2014). These epigenetic mechanisms of thermogenesis are in response to cold stress. As adipose thermogenesis is a key adaptation seen with the implementation of dietary interventions, such as IF and TRF (Hatori et al., 2012; Hatting et al., 2017; Kim K.H. et al., 2017; Li et al., 2017), these epigenetic modulators may be involved in this process, but require further investigation to establish a causal link.

#### Histone Acetylation/Deacetylation

##### Class I/II HDACs

Although direct evidence of adipose class I/II HDAC participation in dietary interventions is currently lacking, histone acetylation and deacetylation in adipose tissues are associated with the beneficial metabolic effects seen with dietary interventions. For example, 30% CR in HFD-fed mice leads to a significant increase in histone 4 acetylation (H4ac) at the *Glut4* promoter, which is associated with increased *Glut4* mRNA expression in WAT and decreased plasma glucose levels (Wheatley et al., 2011). Additionally, a number of HATs and HDACs are involved in regulating adipose thermogenesis, which is one of the primary beneficial mechanisms of dietary interventions (Hatori et al., 2012; Hatting et al., 2017; Kim K.H. et al., 2017; Li et al., 2017). The HATs Gcn5/Pcaf (KAT2A) acetylate H3K9 while CBP/p300 acetylate H3K18 and H3K27 for the activation of BAT-selective genes (e.g., *Pparg*, *Prdm16*, *Angptl4*) (Jin et al., 2011, 2014). In contrast, HDAC1 (class I HDAC) (Li et al., 2016), HDAC3 (class IIa HDAC) (Ferrari et al., 2017b; Liao et al., 2018), HDAC9 (class IIa HDAC) (Chatterjee et al., 2014a,b) and HDAC11 (class IV HDAC) (Bagchi et al., 2018) negatively regulate BAT differentiation and thermogenesis. In separate studies, treatment with an HDAC1 inhibitor (i.e., MS-275) (Ferrari et al., 2017a; Rajan et al., 2018) and genetic ablation of HDAC9 (Chatterjee et al., 2014a,b) and HDAC11 (Bagchi et al., 2018) in HFD-fed mice alleviate the obesity phenotype as a result of reduced body weight (Chatterjee et al., 2014a,b; Ferrari et al., 2017a; Rajan et al., 2018), improved glucose tolerance (Ferrari et al., 2017a; Rajan et al., 2018), and increased thermogenesis and “browning” of WAT (Chatterjee et al., 2014a,b; Ferrari et al., 2017a; Rajan et al., 2018). These metabolic benefits were partially mediated by the hyperacetylation and activation of BAT-selective genes (e.g., *Ucp1*, *Pparg*, *Ppara*, *Prdm16*, *Pgc-1α*, *Cidea*) (Chatterjee et al., 2014a,b; Ferrari et al., 2017b; Bagchi et al., 2018). Therefore, as fasting affects the expression and function of class I/II HDACs in the liver (Mihaylova et al., 2011) and hypothalamus (Funato et al., 2011), it would be promising to explore and identify a causal regulator and mechanism of histone acetylation in fasting- and dietary intervention-mediated adipose tissue remodeling and thermogenesis.

##### Class III HDAC (SIRT1)

Sirtuin 1, a class III HDAC that is upregulated in WAT of mice upon CR (Chen et al., 2008) and fasting (Picard et al., 2004), acts as a negative modulator of adipogenesis. SIRT1 complexes with NCoR/SMRT at the *Pparg* promoter to co-repress target genes involved in TG storage (Picard et al., 2004) and also post-translationally deacetylates FOXO1 to increase the expression of its target gene *Atgl* in TG hydrolysis (Chakrabarti et al., 2011).

Another role of SIRT1 is in the attenuation of adipose inflammation, as seen with dietary interventions, such as CR and IF (Wheatley et al., 2011; Kim K.H. et al., 2017; Liu B. et al., 2019). SIRT1, in association with FOXO1 and C/EBPα, forms a transcriptional activator complex at the *Adipoq* promoter (Qiao and Shao, 2006) for increased stimulation of the anti-inflammatory adiponectin upon CR and IF (Zhu et al., 2004; Kim K.H. et al., 2017). SIRT1-dependent deacetylation of *NF-kB* and *IL-1β* promoter sites have also been reported (Yoshizaki et al., 2009; Liu et al., 2011). Similarly, over-expression of SIRT1 (Gillum et al., 2011) and the use of SIRT1 activators (SRT1720, SRT2379, resveratrol) (Yoshizaki et al., 2009; Yoshizaki et al., 2010; Liu et al., 2016) in HFD-fed or genetically obese mice, suppress NF-kB signaling and gene expression (e.g., *IL-6*, *Tnfa*, *Mcp-1*) (Yoshizaki et al., 2009, 2010; Gillum et al., 2011) and reduce macrophage infiltration in WAT (Yoshizaki et al., 2010; Gillum et al., 2011). Altogether, these studies suggest that the anti-inflammatory effects of dietary interventions may be mediated by SIRT1.

Moreover, SIRT1 expression is increased in BAT of mice upon a 48-h fast (Cordeiro et al., 2013) and 40% CR (Wei et al., 2020). Genetic over-expression (Boutant et al., 2015) and activation (SIRT1720) (Feige et al., 2008) of SIRT1 in mice induces BAT thermogenesis and lipid catabolism, which are mediated by increased expression of BAT-selective transcriptional regulators (PPARα, PPARγ, PGC-1α, PGC-1β, FOXO1, FOXO3a), uncoupling and detoxification factors (UCP1, UCP3, SOD1, SOD2) and FAO genes (*Mcad*, *Lcad*, *Cpt1b*, *Cpt1a*) (Feige et al., 2008; Boutant et al., 2015). Additionally, SIRT1 gain-of-function mice exhibit a greater “browning” phenotype of WAT, indicated by the appearance of smaller adipocytes and elevated brown adipocyte marker genes (*Ucp1*, *Dio2*, *Cebpb*, *Cox7a1*, *Cidea*) upon cold exposure, in comparison to wild-type mice. The post-translational SIRT1-dependent lysine deacetylation of PPARγ and its interaction with the browning co-factor Prdm16 allow for this thermogenic remodelling of WAT (Qiang et al., 2012). These studies thereby suggest that the upregulation of SIRT1 may mediate the increased thermogenesis and WAT “browning” seen upon dietary interventions (Hatori et al., 2012; Hatting et al., 2017; Kim K.H. et al., 2017; Li et al., 2017), however further studies are still required to establish a mechanistic link.

##### Class III HDAC (SIRT2)

Sirtuin 2, another class III HDAC, is also upregulated by CR and fasting in WAT (Wang et al., 2007) and can reduce cellular lipid stores and oxidative stress as seen with dietary interventions (Jing et al., 2007; Wang et al., 2007; Wang and Tong, 2009; Perrini et al., 2020). In adipocytes, the post-translational SIRT2-mediated deacetylation of FOXO1 inhibits the transcriptional activation of PPARγ target genes involved in adipogenesis (Wang and Tong, 2009; Perrini et al., 2020). Additionally, SIRT2 can mediate the post-translational deacetylation of FOXO3a to promote the expression of FOXO target genes involved in the reduction of cellular reactive oxygen species (ROS) (*MnSOD*), the apoptotic clearance of damaged cells (*Bim*) and the inhibition of cell proliferation and propagation of mutations (*p27*^*kip1*^) (Wang et al., 2007).

## Epigenetic Regulation in Liver During Metabolic Disease and Dietary Intervention

### The Liver in Health and Metabolic Disease

**FIGURE 4:**
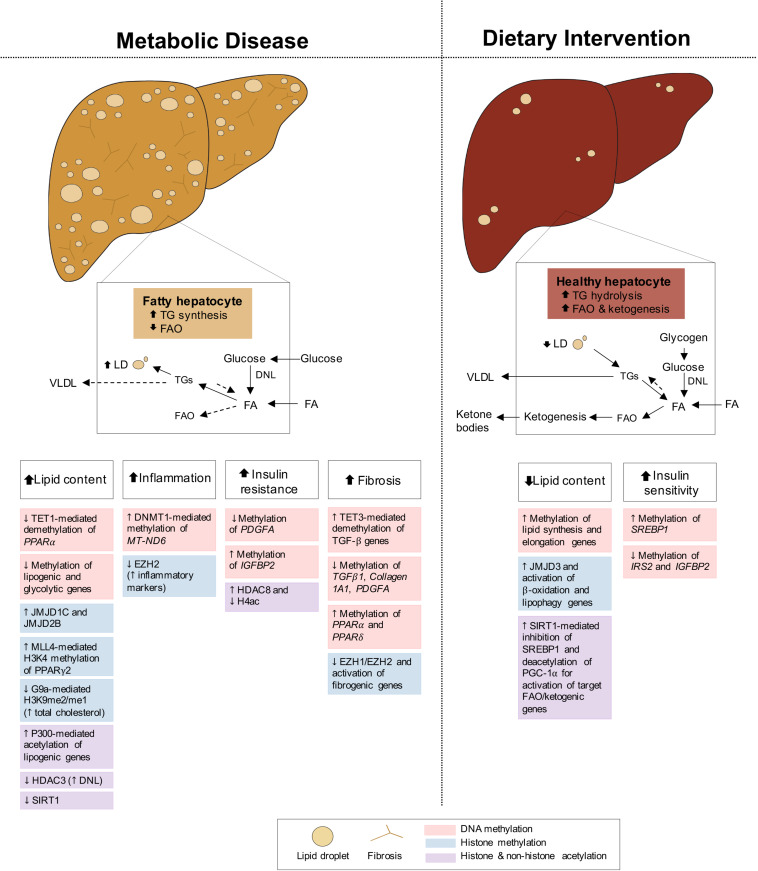
Epigenetic changes of liver in metabolic disease and dietary intervention. Liver in metabolic disease (i.e., NAFLD/NASH) presents with increased lipid content, inflammation, insulin resistance and fibrosis. The liver in dietary intervention has reduced lipid content and improved insulin sensitivity. These physiological differences of the liver can be explained by epigenetic changes involving DNA methylation, histone methylation and histone and non-histone acetylation.

Fatty liver disease is closely associated with both obesity and T2D, with 82% of NAFLD patients presenting with obesity and 48% with T2D, in America (Younossi et al., 2016; Purnell et al., 2017). Intimately linked to systemic energy utilization and storage, the liver functions differently in the fed and fasted states, and its dysregulation can cause NAFLD. During feeding conditions, insulin promotes the storage of glucose into FA and TG or as glycogen through glycogenesis. During fasting, glucagon stimulates the mobilization of TG and glycogen stores for fuel delivery to extra-hepatic tissues, while simultaneously activating hepatic FAO and gluconeogenesis, fueled by adipocyte lipolysis and muscle proteolysis, respectively (Rui, 2014). Under conditions of metabolic dysregulation or disease, insulin resistance of the liver promotes inappropriate upregulation of gluconeogenesis while *de novo* lipogenesis (DNL) pathways remain insulin sensitive, contributing to hyperglycemia and hepatic lipid accumulation, respectively (Brown and Goldstein, 2008; Figure 4). Impaired FAO and very-low-density lipoprotein (VLDL) secretion as a result of insulin resistance further increase fat deposits in the liver (Bhatt and Smith, 2015). NAFLD develops when hepatic lipid stores exceed 5% of tissue mass, leading to increased inflammation, collagen deposition, fibrosis and cell death. If left untreated, NAFLD can progress to non-alcoholic steatohepatitis (NASH) and may continue to cirrhosis and hepatocellular carcinoma (HCC) (Byrne and Targher, 2015; Friedman et al., 2018). Altogether, the metabolic role of the liver in integrating these endogenous and exogenous fuel sources requires constant transcriptional modulation. Below we highlight some epigenetic changes regulating hepatic gene expression in both disease and dietary interventions.

### Epigenetic Changes in the Liver in Metabolic Disease

#### DNA Methylation/Demethylation

Hepatic methylome and transcriptome studies have identified epigenetic links to the differentially expressed genes underlying

the development of hepatic insulin resistance, T2D, and NAFLD (Ahrens et al., 2013; Nilsson et al., 2015; Kirchner et al., 2016; de Mello et al., 2017; Abderrahmani et al., 2018; Gerhard et al., 2018; Hotta et al., 2018). Notably, in the liver of T2D patients, the majority of significant differentially methylated CpG sites show reduced DNA methylation (Nilsson et al., 2015; Kirchner et al., 2016; Abderrahmani et al., 2018). Hypomethylation of a CpG site within or proximal to the activating transcription factor (ATF)-binding motif of hepatic genes accounts for increased expression of these genes involved in glycolysis (*PFKL*), DNL (*ACACA*, *FASN*), and insulin signaling (*PRKCE*) in obese and T2D patients (Kirchner et al., 2016). Moreover, the epigenetic induction of *PDGFA*, which encodes platelet-derived growth factor α (PDGF-AA), appears to be central to hepatic disease progression, as PDGF-AA causes insulin resistance by reducing hepatic insulin receptor density in a protein kinase C (PKC)-dependent manner (Thieringer et al., 2008). In patients with T2D, a CpG site (cg14496282) within *PDGFA* is found to be hypomethylated, leading to increased hepatic *PDGFA* expression and PDGF-AA secretion from insulin-resistant human hepatocytes (Abderrahmani et al., 2018). Additionally, *PDGFA* expression is positively correlated with hepatic fibrosis and NASH risk, and as such, the over-expression of PDGF-AA in mouse liver results in spontaneous liver fibrosis (Thieringer et al., 2008). Since the degree of hepatic fibrosis is an indicator of morbidity and mortality of liver diseases including NAFLD (Dulai et al., 2017), a DNA methylation study conducted with liver samples with NAFLD-related cirrhosis has identified genes enriched in ligand-activated nuclear receptor signaling pathways, involving farnesoid X receptor (FXR), liver X receptor (LXR), and retinoid X receptor (RXR), that play roles in fatty liver disease (Tanaka et al., 2017; Gerhard et al., 2018). Other DNA methylation studies (Ahrens et al., 2013; Nilsson et al., 2015; de Mello et al., 2017; Abderrahmani et al., 2018; Hotta et al., 2018) have also found a number of common differentially methylated sites, including the fibroblast growth factor receptor 2 (*FGFR2*) involved in liver fibrosis (Thieringer et al., 2008). In addition, hypomethylation of CpG sites within *TGFβ1*, *Collagen 1A1*, and *PDGFα* as well as hypermethylation of CpG sites within *PPARα* and *PPARδ* are frequently associated with the increased risk of fibrosis in NAFLD patients (Zeybel et al., 2015; Abderrahmani et al., 2018).

However, the question remains as to why hypomethylation is often found in metabolically dysfunctional livers as well as adipose tissues? Circulating folate levels are reduced in T2D patients compared with non-diabetic subjects (Nilsson et al., 2015). Since folate is a methyl donor in the methylation cycle, hypomethylation in the liver as well as in the pancreas of T2D patients (Dayeh et al., 2014) can be explained by a methyl donor supply consumption imbalance (Zhou et al., 2011; Nilsson et al., 2015). This is supported by a previous study using mice fed with a methyl-deficient diet (Pogribny et al., 2009). Lack of methyl donors accompanied by a loss of genomic cytosine methylation and a change in the expression of hepatic DNA methyltransferases, causes NAFLD and even NASH in mice, highlighting the role of hypomethylation in hepatic steatosis.

While hypomethylation is more common in hepatic tissues of metabolic disease, patients with NASH exhibit higher hepatic expression of DNMT1, which increases methylation and decreases expression of mitochondrial NADH dehydrogenase 6 (MT-ND6), leading to ultrastructural defects in mitochondrial morphology (Pirola et al., 2013). In addition, increased methylation at a CpG site (cg11669516) and reduced gene expression of insulin-like growth factor binding protein 2 (*IGFPB2*) are often found in mice and patients with NAFLD and NASH (Ahrens et al., 2013). Consistently, HFD feeding in young mice induces hypermethylation of *Igfbp2* and reduces its expression prior to diet-induced obesity and hepatic steatosis development. This epigenetic inhibition of *Igfbp2* becomes stable over time in adult mice, suggesting *Igfbp2* methylation as a predictable risk indicator of liver disease development (Kammel et al., 2016). Together, genome-wide DNA methylation studies combined with *ex vivo* and *in vitro* analyses provide key epigenetic mechanisms underlying NAFLD development and progression in obese and T2D patients, by linking differential methylation states with the regulation of hepatic glucose and lipid metabolism, insulin resistance, and hepatic fibrosis.

##### Regulators of DNA methylation

In addition to changes in genomic DNA methylation, differential expression of DNA methylation regulators has been associated with the development of hepatic diseases, particularly the TET proteins involved in hydroxymethylation, the initial step in the reversal of DNA methylation. TET1 expression is reduced in both *in vitro* (HepG2 cells containing FA medium) and *in vivo* (HFD-fed mice) models of NAFLD (Wang et al., 2020). Loss of *Tet1* (*Tet*^–/–^) in mice further exacerbates HFD-induced NAFLD, indicated by increased intrahepatic TG levels. This study suggests that hepatic Tet1-mediated hydroxymethylation of the *PPARα* promoter enhances FAO and thereby prevents NAFLD development.

Furthermore, hepatic fibrosis is a critical pathological process that affects clinical management, as its advancement determines the therapeutic reversibility of NAFLD by leading to irreversible cirrhosis and even HCC (Stal, 2015; Thiele et al., 2017). Abnormal activation of the inflammatory transforming growth factor-beta (TGF-β) signaling pathway along with the transdifferentiation of hepatic stellate cells (HSCs) into proliferative, fibrogenic myofibroblasts, primarily drive hepatic fibrosis through the production of the extracellular matrix (ECM) (Tsuchida and Friedman, 2017). TET3 expression is increased in hepatocytes and HSCs of human fibrotic livers (Xu et al., 2020). In HSCs, TET3 mediates demethylation at specific CpG sites of genes involved in the TGF-β pathway, including *TGFB1*, to promote profibrotic gene expression and subsequent ECM production. In contrast, siRNA-mediated *TET3* knockdown ameliorates liver fibrosis in mice, suggesting its crucial role in the pathological development and progression of hepatic fibrosis. This transdifferentiation of HSCs, largely regulated by a number of epigenetic processes including DNA methylation as described here, and post-translational modification of histones, is reviewed in detail elsewhere (Barcena-Varela et al., 2019).

#### Histone Methylation/Demethylation

The development of metabolic dysfunction in liver diseases accompanies global histone modifications including acetylation and methylation (Nie et al., 2017). Notably, alterations in global histone methylation patterns and expression of the regulators, such as HMTs and HDMs, during development and progression of NAFLD have been reported in a number of recent studies.

##### H3K9 methylation (EHMT2, JMJD1C, JMJD2B, PHF2)

The hepatic expression of G9a (*EHMT2*), a H3K9 HMT, is markedly reduced in genetically induced (i.e., *db/db*) and HFD-fed obese mice (Xue et al., 2018). The liver-specific loss of G9a is associated with a selective decrease in hepatic H3K9me2/me1 and an increase in serum cholesterol levels (Lu et al., 2019). Upon liver injury (e.g., lipopolysaccharide and acetaminophen overdose), G9a mutant mice exhibit severe liver phenotypes associated with increased immune cell infiltration, ROS production and cell death (Zhang et al., 2020), suggesting an epigenetic protective role of G9a in the liver. On the other hand, the H3K9 demethylase, JMJD1C (KDM3), a candidate gene associated with T2D and plasma TG levels (Chasman et al., 2009; Teslovich et al., 2010; Zhang H. et al., 2016), regulates hepatic lipogenic gene expression (e.g., *FAS*, *ACC*, *SREBF1*) by demethylating the H3K9me2/me3 transcriptional repressor marks and leading to increased chromatin accessibility (Viscarra et al., 2020). Over-expression of *JMJD1C* in the liver increases DNL, whereas liver-specific deletion of *Jmjd1c* protects mice from diet-induced hepatic steatosis and insulin resistance. Similarly, the H3K9 di- and tri-demethylase JMJD2B (KDM4B), involved in establishing the H3K9me activation mark, is upregulated in livers of diet-induced obese mice, resulting in increased hepatic PPARγ2 expression and induction of hepatic steatosis (Kim et al., 2018). Moreover, Phf2, another H3K9 HDM, specifically demethylates H3K9me2 on the promoter of carbohydrate-responsive element-binding protein (ChREBP) (Bricambert et al., 2018), a major regulator of glycolytic and lipogenic genes (Ortega-Prieto and Postic, 2019). Interestingly, while glucose homeostasis remains preserved, liver-specific *Phf2* over-expression results in hepatosteatosis, mediated by increased stearoyl-CoA desaturase (Scd1) expression and accumulation of monounsaturated fatty acids. Conversely, *Phf2* silencing leads to liver fibrosis upon a high-fat, high-sucrose diet. With supporting human data, this study suggests Phf2 as a targetable epigenetic checkpoint to prevent NAFLD progression (Bricambert et al., 2018). Together, these studies demonstrate the critical and dynamic implications of H3K9 HMTs and HDMs in the development and progression of liver diseases.

##### H3K27 methylation (EZH1/EZH2)

Another HMT EZH2, which catalyzes trimethylation of H3K27 (H3K27me3) for transcriptional repression, also plays a key role in liver diseases. EZH2 expression is reduced in the liver of NAFLD rats and FA-treated HepG2 hepatocytes and is inversely correlated with lipid accumulation and inflammatory marker expression (Vella et al., 2013). The steatosis-related phenotypes are recapitulated when treated with 3-Deazaneplanocin A, an EZH2 inhibitor, suggesting a causal role of EZH2 in NAFLD development. In addition, EZH1, a homolog of EZH2, has H3K27 methyltransferase activity and can partially compensate for the loss of EZH2 (Ezhkova et al., 2011). Notably, when both *Ezh1* and *Ezh2* are deficient in the liver, the mutant mice develop liver fibrosis with increased fibrogenic gene expression (*Fstl1*, *Fbn1* and *Col1a1*) (Grindheim et al., 2019).

##### H3K4 methylation (MLL4)

While PPARγ2 is a master transcriptional factor of adipogenesis in adipocytes, its expression is elevated in the fatty livers of obese animal models and NAFLD patients (Vidal-Puig et al., 1996; Westerbacka et al., 2007). Hepatic PPARγ2 stimulates the uptake and re-esterification of FA into lipid droplets by upregulating *Cd36*, *Fabp4*, *Mag*, *Plin2*, and *Fsp27*/*Cidec*, and thereby promoting steatosis (Kim et al., 2016; Kim K. et al., 2017). This upregulation of hepatic PPARγ2 can be epigenetically achieved by H3K4 methyltransferase MLL4 (KMT2D) (Kim et al., 2016) as well as through defective HDAC3, which normally associates with retinoic acid receptor-related orphan receptor alpha (RORα) to repress PPARγ2 transcription (Kim K. et al., 2017). Together, these studies provide multimodal histone modulatory mechanisms of NAFLD via methyltransferase- and deacetylase-mediated transcriptional regulation of hepatic PPARγ2.

#### Histone Acetylation/Deacetylation

The modulation of hepatic histone acetylation, achieved through HATs and HDACs, contributes to the development of NAFLD. Among HATs, the transcriptional coactivator p300 is activated in mouse models of obesity and T2D, leading to post-translational hyperacetylation of ChREBP, involved in the transcriptional activation of lipogenic (e.g., *Acc*, *Fas*) and glycolytic (e.g., *Pepck*, *G6Pase*) genes (Bricambert et al., 2010). The activity of hepatic p300 is negatively regulated by salt-inducible kinase 2 (SIK2), thus, liver-specific *SIK2* knockdown results in increased transcriptional activity of ChREBP via p300-mediated acetylation. In addition, hepatic p300 over-expression is sufficient to induce NAFLD and insulin resistance. Conversely, inhibition of HAT activity prevents NAFLD: the novel HAT inhibitor, tannic acid, binds to p300 and disrupts its occupancy on lipogenic genes (e.g., *Fasn*, *Acly*), leading to hypoacetylation of H3K9ac and H3K36ac (Chung et al., 2019). Together, these findings suggest a role for acetyltransferase p300 in NAFLD development.

##### Class I/II HDACs

Histone deacetylases also have key implications in the regulation and dysregulation of hepatic metabolism. As a significant metabolic hub, the functions of the liver in lipid, carbohydrate and amino acid metabolism as well as detoxification are regulated throughout the day by circadian clocks (Reinke and Asher, 2016). This rhythmic hepatic metabolism is orchestrated by epigenetic modulation, mainly through HDAC3, which is recruited by a key circadian clock component, Rev-ebrα (Feng et al., 2011). Upon hepatic *Hdac3* depletion, mice develop hepatosteatosis with increased DNL, suggesting a critical role of histone acetylation-mediated circadian changes in the prevention of NAFLD. Additionally, HDAC8 has been identified as a commonly upregulated gene in dietary and genetic obesity-promoted HCC mouse models as well as in human HCC cells and tissues (Tian et al., 2015). HDAC8 promotes insulin resistance as well as cell proliferation, while its knockdown inhibits NAFLD-HCC tumorigenicity. Specifically, HDAC8, in association with the HMT EZH2, epigenetically regulates the Wnt pathway via decreased histone H4 acetylation and increased H3K27me3 methylation. This finding suggests an epigenetic mechanism involving HDAC8 in the progression of NAFLD-associated HCC.

##### Class III HDAC (SIRT1)

Sirtuin 1, a class III HDAC, acts on hepatic metabolic regulators in response to hormonal and nutritional signals. Sirt1 levels are reduced in a HFD-induced NAFLD rodent model (Deng et al., 2007) and are significantly lower in obese patients with severe steatosis (Wu et al., 2014; Mariani et al., 2015). Liver-specific deletion or knockdown of *Sirt1* in mice leads to fatty liver disease even without a HFD challenge (Rodgers and Puigserver, 2007; Wang R.H. et al., 2010; Xu et al., 2010; Yin et al., 2014). Upon HFD or an alcoholic diet, these *Sirt1* mutant mice develop severe liver injury and fibrosis (Purushotham et al., 2009; Yin et al., 2014; Ramirez et al., 2017). Moreover, these mice lacking hepatic *Sirt1* exhibit elevated gluconeogenesis, leading to hyperglycemia and insulin resistance, which suggests that hepatic SIRT1 plays a role in not only protecting the liver from steatosis, but also in maintaining whole-body glucose metabolism.

### Epigenetic Changes in the Liver in Dietary Intervention

Hepatic lipid accumulation is the primary characteristic and key contributor to NAFLD pathogenesis. Several dietary interventions, such as IF, CR, and KD, have shown protection against and improvement in hepatosteatosis with increased FAO, ketogenesis, and reduced lipogenesis in both obese or diabetic mice (Badman et al., 2009; Baumeier et al., 2015; Kim K.H. et al., 2017; Marinho et al., 2019) and humans (Larson-Meyer et al., 2008; Browning et al., 2011; Sevastianova et al., 2012; Yu et al., 2014; Drinda et al., 2019; Johari et al., 2019; Luukkonen et al., 2020). Additionally, in both rodents and humans, dietary interventions prevent and alleviate the onset of hepatic inflammation (Horrillo et al., 2013; Marinho et al., 2019) and fibrosis (Horrillo et al., 2013; Johari et al., 2019), associated with the more severe NASH and cirrhosis condition of the liver (Figure 4). Taken together, the success of dietary interventions in halting and, in some cases, reversing NAFLD progression, makes it a promising alternative to current therapeutics. Thus, an improved understanding of both accompanying and causal epigenetic changes in the prevention and/or treatment of NAFLD will be necessary for determining novel molecular biomarkers and specific pharmaceutical targets for clinical translation.

#### DNA Methylation/Demethylation

Consistent with its anti-aging effect in humans and other model organisms (Fontana and Partridge, 2015; Redman et al., 2018), 40% CR can protect against the age-related increase in global hepatic DNA methylation (Miyamura et al., 1993) and reduce the epigenetic age of the mouse liver by approximately 1.7 years (Maegawa et al., 2017; Wang et al., 2017). In particular, a study comparing young and aged female mice showed that 40% CR delays the epigenetic aging of hepatic lipid metabolism by inducing hypermethylation and down-regulation of key enzymes involved in hepatic insulin resistance (e.g., *Srebp1*), lipid synthesis (e.g., *Acly*, *Mel*, *Aacs2*, *Acac*, *Pklr*, *Gpam*) and lipid elongation (e.g., *Elov15*, *Elov16*). These changes increase insulin sensitivity while reducing lipid content and chain length of TG-associated FA in the livers of old female mice (Hahn et al., 2017). More specifically, the reduction in the level of FA elongases and the subsequent shift in the hepatic TG pool from long-to medium-chain TG (Hahn et al., 2017) is associated with the prevention of diet-induced insulin resistance and liver disease (Ronis et al., 2013; Geng et al., 2016). Sterol regulatory element-binding protein 1 (SREBP1) contributes to insulin resistance by interfering with the binding of FOXO1 to the insulin receptor substrate 2 (*Irs2*) promoter, thereby repressing *Irs2* expression. CR has been shown to improve insulin resistance in mice through hypermethylation and downregulation of *Srebp1* as well as through hypomethylation and upregulation of *Irs2*, the direct target of SREBP1 (Hahn et al., 2017). Moreover, in a separate study, CR-treated mice with a 25% reduction of body weight, exhibit decreased methylation and increased expression of hepatic *Igfbp2* – an effect that is abolished by HFD re-feeding (Kammel et al., 2016). Since the reverse, increased methylation and decreased expression of *IGFBP2*, is found in both mice and humans with NAFLD and NASH (Ahrens et al., 2013), this CR-mediated change in *Igfbp2* levels indicates metabolic improvements (Nam et al., 1997; Heald et al., 2006; Carter et al., 2014). CR also regulates the expression of DNA methylation modulators. In both young and old female mice, 40% CR increased *Tet3* and *Dnmt3a* and decreased *Tet2*, *Dnmt1* and *Dnmt3b* expression in the liver (Hahn et al., 2017). While altered hepatic expression of DNMT and TET enzymes in regulating DNA methylation is not well understood, this finding suggests that CR may modulate hepatic transcripts via dynamic regulation of DNA methylation machinery.

#### Histone Methylation/Demethylation

Dietary interventions, such as fasting, can modulate hepatic histone methylation and subsequent gene transcription. In particular, fasting-induced protein expression of JMJD3 (KDM6B), a H3K27me3 HDM (Seok et al., 2018; Byun et al., 2020), stimulates the expression of β-oxidation (e.g., *Fgf21*, *Cpt1a*, *Mcad*) (Seok et al., 2018) and autophagy genes (e.g., *Tfeb*, *Ulkl*, *Atgl*) (Byun et al., 2020) in the liver, thereby promoting the removal of hepatic lipid stores via increased lipolysis and lipophagy. This histone modulation by hepatic JMJD3 in response to fasting is mediated by its association with two transcriptional activating complexes; JMJD3 in complex with PKA-phosphorylated SIRT1 (Ser434) and PPARα (Seok et al., 2018) or PKA phosphorylated JMJD3 (Thr1044) in complex with FGF21 (Byun et al., 2020). The fasting-induced JMJD3-SIRT1-PPARα complex additionally forms a feed-forward regulatory loop, which auto-induces the expression of its genes, including *Fgf21*, *Jmjd3, Sirt1* and *Ppara*, to amplify the cellular responses under fasting conditions. Importantly, the downregulation of *Jmjd3* and its associated factors (*Sirt1, Fgf21, Ppara*) in the mouse liver results in reduced hepatic β-oxidation and increased steatosis. These data suggest a critical role for histone methylation modulators in mediating the metabolic improvements associated with fasting-related dietary interventions against liver metabolic dysfunction.

#### Histone Acetylation/Deacetylation

##### Class I/II HDACs

Hepatic histone acetylation in the context of dietary interventions mainly involve HDACs. Specifically, under fasting conditions, class IIa HDACs upregulate the hepatic gluconeogenic gene program. Fasting-induced glucagon-secretion in primary mouse hepatocytes promotes protein kinase A (PKA)-mediated phosphorylation and inactivation of SIK2, thereby allowing for the nuclear translocation of unphosphorylated class IIa HDACs (Wang et al., 2011). Nuclear HDAC4 and HDAC5 (class IIa HDACs) in association with HDAC3 (class I HDAC) deacetylate and activate FOXO transcription factors for the induction of gluconeogenic genes (Mihaylova et al., 2011).

##### Class III HDACs (SIRT1)

Under low nutrient conditions (i.e., IF, CR), an increase in the NAD^+^/NADH ratio activates hepatic SIRT1, a class III HDAC. The genetic over-expression of *SIRT1* or its activation by resveratrol treatment protects against HFD-induced hepatosteatosis and glucose intolerance (Rodgers and Puigserver, 2007; Pfluger et al., 2008) as well as alcoholic diet-induced liver injury and fibrosis (Ajmo et al., 2008; Ramirez et al., 2017). Mechanistically, SIRT1 post-translationally deacetylates and activates PGC-1α, which interacts with its co-factor HNF4α to stimulate the expression of gluconeogenic (e.g., *G6pase, Pepck, Fbp1, G6pc*) and β-oxidation (e.g., *Mcad, Cpt-1a, Dgat2*) genes, while repressing glycolytic genes (e.g., *Lpk, Gck*) (Nemoto et al., 2005; Rodgers et al., 2005; Rodgers and Puigserver, 2007). SIRT1-deacetylated PGC-1α also regulates PPARα target genes involved in hepatic FAO and ketogenesis (Rodgers and Puigserver, 2007; Purushotham et al., 2009; Hayashida et al., 2010). Hepatocyte-specific deletion of *Sirt1* results in the hyperacetylation of PGC-1α at PPAR response element (PPRE) sites on target genes, thereby inhibiting PPARα signaling (Purushotham et al., 2009). Additionally, fasting-induced hepatic SIRT1 can post-translationally deacetylate SREBP1 at its DNA-binding domain, leading to its ubiquitin-mediated proteasomal degradation (Hirano et al., 2001; Sundqvist and Ericsson, 2003; Walker et al., 2010). Inhibition of hepatic SREBP activity promotes fat mobilization through the activation of lipolytic and FAO pathways, thereby reducing hepatic fat stores and protecting against hepatic steatosis (Walker et al., 2010). SIRT1 also mediates the acetylation of histones H3 and H4 (i.e., H3K9Ac, H3K56Ac, H3K18Ac and H4K16Ac) (Bosch-Presegue and Vaquero, 2015), for the regulation of chromatin structure and transcriptional activation.

However, the necessity of SIRT1 in IF, TRF, and FMD has not been mechanistically tested yet. Interestingly, a study comparing transcriptomic changes by IF (i.e., ADF) and *Sirt1* over-expression in mice concluded that despite functional similarities such as improved insulin sensitivity, *Sirt1* gain-of-function does not mimic nor boost the metabolic effects of IF (Boutant et al., 2016). This suggests that Sirt1 may not be the only mediator of fasting-involved dietary interventions. Moreover, the current literature has conflicting results in regard to hepatic SIRT1 expression under fasting and nutritional interventions (i.e., CR). Although most studies show an upregulation of hepatic SIRT1 upon fasting or CR (Cohen et al., 2004; Rodgers et al., 2005; Deng et al., 2007; Rodgers and Puigserver, 2007; Hayashida et al., 2010; Ding et al., 2017), some studies have reported a decrease (Chen et al., 2008) or no change (Barger et al., 2008) in expression. In particular, it was reasoned that the decreased level and activity of Sirt1 in the livers of CR mice was due to low cellular NAD^+^/NADH levels (Hagopian et al., 2003; Lin et al., 2004; Chen et al., 2008; Kawakami et al., 2012). Furthermore, the liver-specific down-regulation of *Sirt1* in these studies resulted in reduced hepatic fat synthesis and improved glucose homeostasis (Chen et al., 2008; Erion et al., 2009) – an effect that contradicts other studies showing increased hepatic FA and cholesterol, impaired glucose tolerance, hepatic inflammation and steatosis (Rodgers and Puigserver, 2007; Purushotham et al., 2009) upon *Sirt1* deficiency. Aside from variation in animal species, strain or age used in the studies, differences in hepatic SIRT1 expression may also be attributed to differences in length of fasting or extent of food restriction. Particularly, SIRT1 has been shown to be upregulated in the liver of mice upon long-term fasting (18−24 h), but not short-term fasting (6−8 h), and thus may play a role in the later stages of nutrient depletion (Liu et al., 2008). Some discrepancies might also originate from different experimental settings. Although metabolic phenotyping (e.g., indirect calorimetry) and tissue harvesting are commonly performed after an overnight fast post-feeding day, the metabolic benefits of dietary interventions can take place during fasting and/or refeeding. For example, both the elevation of energy expenditure via adipose thermogenesis by IF (Kim K.H. et al., 2017) and pancreatic β-cell regeneration by FMD (Cheng et al., 2017) occur during the refeeding period after fasting and nutritional interventions. Regardless, these conflicting studies do not undermine the importance of SIRT1 in the liver and the use of SIRT1 agonists or fasting-like mimetics as possible therapeutic options for patients with metabolic syndrome. Overall, the liver-specific epigenetic mechanisms described herein are important in understanding the pathophysiological development of NAFLD and its possible alleviation via dietary interventions.

## Epigenetic Regulation in Pancreas During Metabolic Disease and Dietary Intervention

### The Pancreas in Health and Metabolic Disease

**FIGURE 5:**
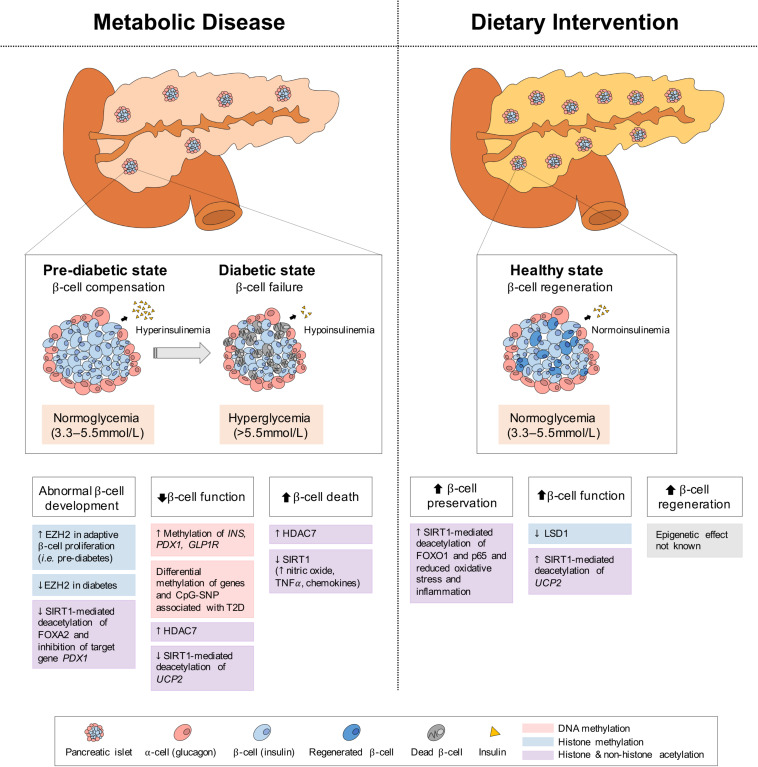
Epigenetic changes of pancreas in metabolic disease and dietary intervention. In metabolic disease (i.e., T2D), the initial proliferation of pancreatic β-cells increase insulin secretion, but eventual β-cell failure leads to hypoinsulinemia and hyperglycemia. In dietary intervention, pancreatic β-cells are preserved through reduced inflammation and oxidative stress and present with increased function (i.e., GSIS) and regeneration. These physiological differences in the pancreas can be explained by epigenetic changes involving DNA methylation, histone methylation and histone and non-histone acetylation.

The pancreas is a secretory organ with both exocrine (acinar) and endocrine (islet) function. Among the pancreatic islet cell types, the glucagon-secreting α-cells and the insulin-secreting β-cells are primarily involved in regulating the metabolic pathways of the fed and fasted states. Under fasting conditions, glucagon is released to increase blood glucose levels through the promotion of hepatic gluconeogenesis and glycogenolysis, whereas under feeding conditions, insulin is secreted to promote the uptake of glucose, amino acids and FA and to stimulate processes of glycogenesis, protein synthesis and DNL in insulin-sensitive metabolic tissues (Piciucchi et al., 2015; Roder et al., 2016; Weisbeck and Jansen, 2017). In the setting of insulin resistance, β-cells increase their insulin secretion (i.e., hyperinsulinemia) to maintain normal glucose levels (Johnson and Alejandro, 2008; Figure 5). However, when β-cells can no longer sustain the increased demand (i.e., hypoinsulinemia), glucose levels rise and initially present as impaired glucose tolerance (Kahn et al., 2014). As β-cell dysfunction progresses, hyperglycaemia and diabetes arise (Weisbeck and Jansen, 2017). Eventually, the hyperactivity of β-cells and high levels of blood glucose and lipids contributing to glucotoxicity and lipotoxicity, can stimulate β-cell apoptosis and further propagate the pathogenesis of T2D (Deng et al., 2010). In addition to the genetic component, T2D is also largely influenced by environmental stressors, such as prolonged physical inactivity and an unhealthy diet (i.e., fatty foods high in dioxins) (Hoyeck et al., 2020; Ibrahim et al., 2020), which result in changes in metabolic gene expression, mediated through epigenetics. Herein we discuss some of the epigenetic changes involved in both T2D development and alleviation via dietary interventions in the pancreas; the key organ regulating both plasma insulin and glucose levels.

### Epigenetic Changes in the Pancreas in Metabolic Disease

#### DNA Methylation/Demethylation

As pancreatic islets are central to T2D development, the methylation status of the promoters of critical genes in islet function and development have been investigated. In islets of T2D patients, the increased DNA methylation of the insulin gene promoter (*INS*) at 4 CpG sites, correlates negatively with insulin mRNA levels and positively with glycated hemoglobin HbA(1c) levels, which reflect the cumulative blood glucose concentration (Yang et al., 2011). Similarly, in an *in vitro* model of a rat β-cell line (INS 832/13), 72-h of high glucose exposure (16.7 mmol/L), which recapitulates conditions of hyperglycemia in T2D, increases DNA methylation within the *Ins* promoter (Yang et al., 2011). Likewise, in another study, 48-h of high glucose exposure (19 mM) in isolated human islets results in differential methylation and expression of genes involved in islet function, including *GLRA1*, *RASD1*, *VAC14*, *SLCO5A1*, *CHRNA5*, and *PDX1*, and a decrease in glucose-stimulated insulin secretion (GSIS) (Hall et al., 2018). A 48-h exposure to palmitate (1 mM), a saturated fatty acid, also reduces GSIS in human islets while inducing methylation changes and differential expression of 290 genes, including the *TCF7L2* and *GLIS3*, markers of T2D risk (Hall et al., 2014). These methylation changes in islets upon high glucose and lipid exposures may be analogous to the effects of glucotoxicity and lipotoxicity in pre-diabetic and diabetic conditions. Moreover, the expression of insulin promoter factor 1 (*PDX1*), a master transcriptional regulator of β-cell development and function, is reduced with increased methylation in pancreatic islets of T2D patients, which also corresponds to increased HbA1c levels (Yang et al., 2012). Similarly, the expression of the glucagon-like peptide-1 receptor (*GLP1R*), involved in enhanced GSIS upon GLP-1 peptide binding (Muller et al., 2019), is decreased in pancreatic islets of T2D patients and hyperglycemic rats (Xu et al., 2007; Shu et al., 2009; Taneera et al., 2012). In particular, the methylation level of a CpG site within the *GLP1R* promoter correlates negatively with its gene expression, but positively with BMI and HbA1c (Hall et al., 2013), suggesting that the obesogenic and diabetic conditions can impact the DNA methylation profile of *GLP1R* and possibly lead to changes in pancreatic β-cell insulin secretion (Hall et al., 2013).

GWAS have identified multiple *loci* associated with the T2D risk and accounting for ∼10% of heritable diabetes (Voight et al., 2010). These include *loci* related to impaired insulin secretion and insulin sensitivity (Ruchat et al., 2009). Additionally, among 40 SNPs previously associated with T2D in human pancreatic islets (McCarthy, 2010), 19 of them (48%) are involved in either introducing or removing a CpG site (Dayeh et al., 2013). Importantly, these CpG-SNP sites are differentially methylated and result in changes in gene expression, alternative splicing events and hormone secretion in human islets. Altered expression of genes associated with T2D risk include *TCF7L2*, *HHEX*, *CDKN2A*, *SLC30A8*, *CDKAL1*, *ADCY5*, and *FS1* (Ruchat et al., 2009; McCarthy, 2010). Moreover, in a separate study, genome-wide DNA methylation quantitative trait locus (mQTL) analysis in human pancreatic islets (Olsson et al., 2014) has identified over 67,000 CpG-SNP pairs, with several mQTLs associated with differential expression of T2D- and insulin secretion-related genes (e.g., *ADCY5*, *KCNJ11*, *INS*, *PDX1* and *GRB10*) in human islets. Together, these studies demonstrate that DNA methylation may provide a causal link between SNPs and pancreatic gene function, thereby contributing to T2D development and progression (Dayeh et al., 2013).

Men are at higher risk for T2D than women (Kautzky-Willer et al., 2016). In isolated islets, GSIS is greater in female versus male donors, independent of β-cell number (Sharp et al., 2011). Differences in the DNA methylome also exist in pancreatic islets between men and women; specifically, significant sex-specific differences have been observed in 61 X-chromosome genes and 18 autosomal genes, including *NKAP*, *SPESP1* and *APLN*, which are expressed at lower levels in female islets. The methylation of *NKAP* and *SPESP1* promoters decreases their expression, and the silencing of *Nkap* and *Apln* in clonal cells reduces GSIS (Sharp et al., 2011), suggesting that differential DNA methylation may explain sex differences in insulin secretion and T2D risk.

#### Histone Methylation/Demethylation

Compared to the implications of DNA methylation in pancreatic metabolic conditions, our understanding of histone modifications in the onset and progression of diseases is limited. Yet, as many studies focus on the regeneration of pancreatic β-cells for the treatment of T2D, histone methylation via transcriptional programming in pancreatic development and function have been studied. While insulin-secreting β-cells and glucagon-secreting α-cells have different physiological functions, a study revealed that human α, β, and exocrine cells share similar profiles of histone methylation of H3K4me3 and H3K27me3, suggesting an epigenomic plasticity of islet cells and their reprogrammable potentials to treat diabetes (Bramswig et al., 2013). For example, the activation of the S6K1 kinase promotes α to β-cell transition by activating β-cell genes while repressing α-cell genes through histone methylation of the activating H3K4me3 and repressing H3K27me3, respectively (Yi et al., 2018). Human pancreatic α-cells can also be reprogrammed into insulin-producing cells by PDX1, and when transplanted, can treat diabetic mice (Furuyama et al., 2019). Pancreatic β-cell proliferation and expansion are highly active early in life in humans and mice (Meier et al., 2008) and decays with maturation and aging (Teta et al., 2005). Interestingly, expression of *EZH2*, a HMT of H3K27me3 and a key regulator of cell differentiation and growth, also decreases with aging in mouse pancreatic β-cells, whereas its expression is increased with adaptive β-cell proliferation after streptozotocin-mediated β-cell destruction (Chen et al., 2009). EZH2 can regulate β-cell proliferation by epigenetically repressing the *Ink4a*/*Anf* locus, which encodes the cyclin-dependent kinase inhibitor p16INK4a and tumor suppressor p19Arf (Chen et al., 2009). Consequently, mice lacking *Ezh2* in pancreatic β-cells exhibit mild diabetes, suggesting a role for pancreatic Ezh2 in β-cell function.

#### Histone Acetylation/Deacetylation

Altered pancreatic histone acetylation plays a part in the development of T2D. Specifically, the expression of *HDAC7*, a class IIa HDAC, is increased in pancreatic islets from patients with T2D (Daneshpajooh et al., 2017) and over-expression of *Hdac7* in rat islets and clonal β-cells reduces insulin content and increases apoptosis, leading to impaired GSIS of β-cells (Daneshpajooh et al., 2017). Conversely, treatment with a class II HDAC inhibitor (MC1568) rescues the dysfunctional insulin release of *Hdac7*-over-expressed β-cells and human islets from T2D donors (Daneshpajooh et al., 2018). Interestingly, the over-expression of *HDAC7* in islets from T2D patients is likely mediated by hypomethylation of HDAC7’s CpG site (Dayeh et al., 2014), suggesting a cooperative epigenetic action.

Pancreatic β-cell SIRT1 (Ramsey et al., 2008) and its genetic polymorphisms are associated with the development of T2D (Dong et al., 2011; Maeda et al., 2011). Additionally, a *SIRT1* mutation (L107P), which mildly reduces HDAC activity, has been found in type 1 diabetes (T1D) patients and leads to hyperinflammation with elevated expression of nitric oxide, cytokines (i.e., TNFα), and chemokines in a β-cell line (MIN6) (Biason-Lauber et al., 2013). Pancreas-specific *Sirt1*-deficient mice (*Pdx1-Cre; Sirt1^*flox/flox*^*) present with glucose intolerance and impaired GSIS of β-cells (Luu et al., 2013; Wang R.H. et al., 2013; Pinho et al., 2015). SIRT1-mediated deacetylation and subsequent repression of *Ucp2* normally activates GSIS (Tordjman et al., 2002; Bordone et al., 2006; Chan and Kashemsant, 2006; Brun et al., 2015), however, the absence or reduction of pancreatic SIRT1 results in increased acetylation and expression of *Ucp2* (Bordone et al., 2006) and other downstream target genes, such as *Pgc-1α*, *Pparγ* (Luu et al., 2013), and *Pparα* (Maiztegui et al., 2018), leading to decreased GSIS. Similarly, upon high glucose exposure (Brun et al., 2015), or the addition of sucrose (10%) to a normal diet (Maiztegui et al., 2018), pancreatic SIRT1 expression is decreased, while *Ucp2* and *Pparα* expression is increased in human and rodent islets, leading to reductions in insulin content and GSIS of β-cells (Brun et al., 2015; Maiztegui et al., 2018). Moreover, as FOXA2 activation by post-translational SIRT1-mediated deacetylation stimulates the expression of its target gene *Pdx1*, essential for pancreatic β-cell development and maturation, SIRT1 insufficiency reduces β-cell formation (Wang R.H. et al., 2013). Furthermore, the age-related decline in SIRT1 activity and the accompanying decrease in GSIS from diminished NAD^+^ biosynthesis suggest an association with age-related metabolic diseases, including T2D (Ramsey et al., 2008). Collectively, these data indicate that genetic and dietary components can have profound effects at the epigenetic level, contributing to β-cell dysfunction and T2D development.

### Epigenetic Changes in the Pancreas in Dietary Intervention

Caloric restriction treatment (30−50%) in rodent models of diabetes (i.e., *db/db*, aged mice or Zucker diabetic fatty rat) improves glucose tolerance and insulin sensitivity (Colombo et al., 2006; Kanda et al., 2015; Sheng et al., 2016; Rosa et al., 2018), β-cell mass (Ohneda et al., 1995; Bates et al., 2008; He et al., 2012; Kanda et al., 2015) and insulin secretion (Ohneda et al., 1995; Colombo et al., 2006; He et al., 2012; Kanda et al., 2015; Figure 5). These physiological benefits are accompanied by a reduction in the expression of genes related to oxidative and ER stress (i.e., *Nox1*, *Chop10*, *Tnfa*, *Sod*, *Cat*, *Gpx1*) (He et al., 2012; Kanda et al., 2015). In particular, isocaloric 2:1 IF in genetically obese (i.e., *ob/ob*) mice improves glucose homeostasis with increased postprandial insulin secretion, particularly GSIS (Kim Y.H. et al., 2019). As IF increases plasma GLP-1 levels, this suggests a possibility of an incretin-mediated insulinotropic effect of dietary interventions. Moreover, a FMD in diabetic (i.e., *db/db*) mice confers improvements in β-cell function as indicated by decreased plasma glucose and increased plasma insulin levels, as well as a reduction in insulin resistance (Cheng et al., 2017). Notably, this study demonstrated that a FMD protects against β-cell failure in late-stage T2D by promoting regeneration of insulin-producing β-cells from Ngn3^+^ pancreatic progenitor cells, particularly during the re-feeding period. These improvements in β-cell development and function are also seen in T2D patients on CR (Malandrucco et al., 2012; Jackness et al., 2013; Sathananthan et al., 2015).

The limited studies investigating the epigenetic effects of dietary interventions in the pancreas mainly pertain to histone acetylation changes. According to one study, 6 days of TRF (12-h fasting/feeding) in mice enhances GSIS in isolated islets without affecting body weight (Wortham et al., 2019) and is accompanied by histone acetylation of pancreatic islets during the re-feeding period. This epigenetic modulation takes place at sites occupied by the HDM LSD1 (KDM1A), which silences enhancers by removing mono- and dimethyl marks from H3K4 and is implicated in pancreatic endocrine cell development (Vinckier et al., 2020). The β-cell specific loss of *Lsd1* results in histone hyperacetylation accompanied by insulin hypersecretion, indicating that the adaptive insulin secretory response to dietary interventions is regulated by the modulation of the epigenome in the pancreas (Rosen et al., 2018).

Another important epigenetic factor mediating the pancreatic response to dietary interventions is SIRT1. CR increases and activates SIRT1 in β-cells, thereby promoting pancreatic insulin secretion and ameliorating the T2D phenotype (Liang et al., 2009; Chen et al., 2010; Deng et al., 2010). Similar to the metabolic benefits conferred by dietary interventions (Chen et al., 2010, 2013), *Sirt1* over-expression in mice preserves glucose homeostasis through improvements in insulin secretion and glucose tolerance (Moynihan et al., 2005; Ramsey et al., 2008). Mechanistically, pancreatic SIRT1-mediated repression of *Ucp2* increases cellular ATP to promote vesicular exocytosis and release of insulin from β-cells (Moynihan et al., 2005; Bordone et al., 2006; Chan and Kashemsant, 2006; Ramsey et al., 2008). Additionally, to accommodate CR-mediated increases in insulin secretion, β-cells proliferate and increase their mass, resulting in larger pancreas size (Chen et al., 2013) – an effect that is also seen with SIRT1 activation (Wu et al., 2019). CR also lowers pancreatic inflammation and oxidative stress (Deng et al., 2010; Lanza-Jacoby et al., 2013; Harvey et al., 2014), which can otherwise lead to β-cell death and failure associated with T2D pathogenesis. This is likely mediated by SIRT1 activation since *SIRT1* over-expression or resveratrol (CR-mimetic) treatment post-translationally deacetylate both p65 for inhibition of the NF-kB inflammatory signaling pathway (Lee et al., 2009) and FOXO1 for defense against oxidative stress (Kitamura et al., 2005; Zhang T. et al., 2016). While limited in number, studies investigating epigenetic changes with dietary interventions in the pancreas highlight key factors and mechanisms involved in ameliorating the T2D condition, and warrant further research on this topic.

## The Metabolic and Epigenetic Interplay Between Gut Microbiota and Diet

The gut is host to trillions of microorganisms, including bacteria, archaea, viruses, and eukaryotes. The combined genome (∼150 times larger than the human genome) and the function of these microorganisms make up the microbiome (Ley et al., 2006a; Qin et al., 2010). While the microbiome influences digestion, gut-hormone secretion, intestinal immunity and inflammation, it is also largely shaped by diet; specifically, diet alterations account for 57% of the changes in gut microbiota populations, whereas genetic mutations only account for 12% (Zhang C. et al., 2010). Cumulating evidence places the gut microbiome and its metabolites at the origin of diet-induced metabolic dysregulation (Ley et al., 2006b), therefore, positive modulations of the gut microbiota by dietary interventions are of therapeutic interest.

**FIGURE 6:**
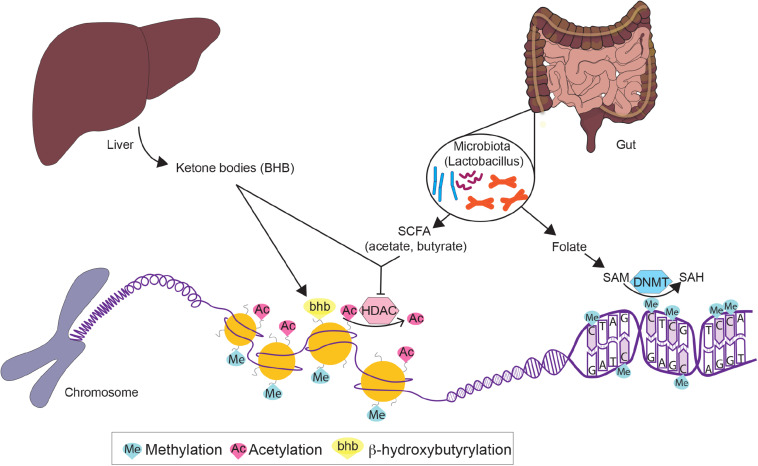
Epigenetic modulation by dietary intervention-induced ketogenesis and gut microbial metabolites. Dietary interventions stimulate ketone body production such as β-hydroxybutyrate, which can modulate gene expression through histone modification (bhb) and inhibition of histone deacetylases (HDACs). Dietary interventions also modulate the gut microbiota, through the release of short-chain fatty acids (SCFA) acetate and butyrate which inhibit HDACs, and folate which provides methyl donors for DNA methyltransferase (DNMT) activity.

Gut microbiota influences the host metabolism through various microbial-derived metabolites, which induce epigenetic alterations of key genes involved in the initiation and progression of diseases (Figure 6). Metabolites, such as folate, choline, vitamin B12, and betaine, can function as methyl donors and participate in DNA methylation processes via the generation of S-adenosylmethionine (SAM). In addition, the gut microbiota ferments complex carbohydrates from the diet to produce small organic acids, most of which are short-chain fatty acids (SCFA) (<95%), such as butyrate, propionate, acetate and lactate (den Besten et al., 2013). These SCFA, particularly butyrate and acetate, inhibit HDACs, leading to transcriptional activation via increased histone acetylation. Additional epigenetic roles of the gut microbiome are well discussed elsewhere (Sharma et al., 2019).

Importantly, fasting-feeding cycles can directly impact the gut microbiota (Thaiss et al., 2014). In particular, dietary intervention-mediated (i.e., IF, FMD, CR, KD) remodeling of gut microbial populations confers various health benefits (Li et al., 2017; Beli et al., 2018; Cignarella et al., 2018; Fabbiano et al., 2018; Wang et al., 2018; Rangan et al., 2019; Ang et al., 2020; Liu et al., 2020). The causal and functional roles of these favorable microbes in dietary interventions are supported by microbiota transplantation and elimination (e.g., antibiotic treatment) studies. For example, IF-induced WAT browning and associated metabolic benefits are abolished in microbiota-depleted mice and subsequently restored with IF-microbiota transplantation (Li et al., 2017). This is in part mediated by the expansion of the *Lactobacillus* bacteria population, resulting in increased levels of serum lactate (Vergnes and Reue, 2014) and acetate (Hernandez et al., 2019), which are taken up by the upregulated monocarboxylate transporter 1 (*Mct1*) in WAT (Iwanaga et al., 2009; Carriere et al., 2014; Li et al., 2017). *Lactobacillus*, populated upon IF, CR and FMD, is a probiotic bacterium with health-promoting properties against metabolic diseases (Sharma et al., 2019) and is involved in the production of folate and the fermentation of pyruvate to acetate and lactate. IF can also increase plasma butyrate levels through greater butyrate-producing *Odoribacter* (Liu et al., 2020). Notably, these health benefits of IF can be achieved through the direct administration of SCFA (Liu et al., 2020), suggesting that metabolites from a re-established microbiome mediate the health benefits of dietary interventions. Although studies of dietary intervention, such as CR and KD, have shown epigenetic-mediated mechanisms of improved gut stem cell homeostasis (Igarashi and Guarente, 2016; Cheng et al., 2019), the association with gut microbiota is still not fully understood and thus warrants further research.

## Ketone Bodies as Epigenetic Regulators in Dietary Intervention

Many of the benefits related to fasting and nutritional interventions, such as IF, CR, FMD and KD, may stem from the activation of the ketogenic pathway and the increased production of ketone bodies (i.e., ketosis). This metabolic switch to ketosis, associated with improvements in lipid and glucose metabolism, contributes to a healthier metabolic state of the tissues discussed in this review. Dysregulation or insufficiency of ketogenesis, by contrast, can be associated with hepatic metabolic abnormalities and may contribute to NAFLD (Cotter et al., 2014; Mannisto et al., 2015; d’Avignon et al., 2018; Fletcher et al., 2019) and liver fibrosis (Puchalska et al., 2019).

Fasting stimulates hepatic ketogenesis to increase systemic ketone body levels. Mitochondrial 3-hydroxy-3-methylglutaryl-CoA synthase 2 (HMGCS2) is the rate-limiting enzyme of the ketogenic pathway and catalyzes the conversion of acetoacetyl-CoA to HMG-CoA – the first step in β-hydroxybutyrate (BHB) synthesis. *HMGCS2* is a downstream target gene of FOXA2, a key transcription factor of hepatic lipid metabolism. Under feeding conditions, insulin/PI3K/Akt-mediated phosphorylation of FOXA2 reduces its transcriptional activity by nuclear exclusion (Wolfrum et al., 2004; von Meyenn et al., 2013; Newman and Verdin, 2014). Conversely, under fasting conditions, glucagon signaling inhibits SIK2 kinase and allows for p300-mediated post-translational acetylation and activation of FOXA2, resulting in the induction of *HMGCS2* transcription (von Meyenn et al., 2013; Newman and Verdin, 2014). In addition to the SIK2-p300-FOXA2 axis, SIRT3, the mitochondrial class III HDAC, directly increases the enzymatic activity of HMGCS2 under fasting conditions through post-translational deacetylation of lysine residues (Shimazu et al., 2010; Newman and Verdin, 2014). Mice lacking *Sirt3* exhibit a reduction in fasting-induced BHB production, indicative of hypoketonemia, along with impaired IF-mediated neurological improvements (Liu Y. et al., 2019).

Acetone, acetoacetate (AcAc) and BHB are the three ketone bodies produced by the liver, from which AcAc and BHB are transported primarily to skeletal muscle and the brain as carriers of additional energy, while acetone is mainly released through exhalation. Among the three ketone bodies, BHB is the most abundant in mammals. Notably, recent studies have demonstrated that ketone bodies play a pivotal role as direct or indirect signaling mediators of cellular and metabolic functions, including epigenetic gene regulation (Newman and Verdin, 2014, 2017; Puchalska and Crawford, 2017). Similar to butyrate (Candido et al., 1978; Cousens et al., 1979; Louis and Flint, 2009), BHB inhibits HDACs, particularly HDAC 1, 3, and 4 (class I and IIa) (Shimazu et al., 2013; Figure 6). Both HDAC3 and HDAC4 are responsible for stimulating the expression of gluconeogenic genes (Mihaylova et al., 2011), hence their inhibition by BHB would result in a reduction in plasma glucose levels, as seen in *Hdac3*-deficient mice (Knutson et al., 2008). In addition, ketosis stimulated by either BHB treatment, 40% CR or overnight fasting in cells and mice all result in histone hyperacetylation, particularly at H3K9 and H3K14, thereby promoting the expression of genes, such as *Foxo3a* – a core regulator of cellular homeostasis (i.e., cell cycle progression), stress response, and longevity induction (Shimazu et al., 2013). Since both butyrate treatment and HDAC inhibition improve hepatic steatosis (Endo et al., 2013; Zhou et al., 2017, 2018) and glucose homeostasis (Gao et al., 2009; Xiao et al., 2011; Cho et al., 2018; Lee et al., 2018) in both mice and humans, enhancing BHB levels through dietary interventions may provide similar beneficial effects.

Furthermore, elevated BHB levels result in increased histone lysine β-hydroxybutyrylation (kbhb), a novel type of histone post-translational modification (Xie et al., 2016; Figure 6). BHB produced from ketogenesis can be complexed with free molecules of Coenzyme A to form BHB-CoA, the donor for kbhb histone modifications (Sabari et al., 2017). A total of 44 histone kbhb sites have been determined in human embryonic kidney cells (HEK293) and mouse liver. These kbhb marks are found in the promoter sites of target genes and result in transcriptional activation. In particular, kbhb on H3K9 (H3K9bhb) is found in fasted liver (Xie et al., 2016). The H3K9bhb acylation mark targets and leads to the upregulation of genes involved in amino acid catabolism, redox balance and circadian rhythm – mediating these specific processes in the switch from feeding to fasting state (Xie et al., 2016). It is thus tempting to speculate that the metabolic benefits by ketogenic dietary interventions, such as IF, CR, FMD and KD, involve histone kbhb-mediated epigenetic modulation.

Pharmaceutical therapeutics stimulating a state of ketosis similar to that with dietary interventions, such as sodium-glucose co-transporter 2 (SGLT2) inhibitors, are being clinically used in the treatment of NAFLD and T2D (Polidori et al., 2018). SGLT2 inhibitors have shown metabolic improvements in hyperglycemia, adiposity, oxidative stress and inflammation (Komiya et al., 2016; Scheen, 2019). While it has not been clearly understood, increased circulating ketone bodies by SGLT2 inhibitors has been considered one of the mechanisms of action mediating the metabolic benefits (Prattichizzo et al., 2018; Wojcik and Warden, 2019). In addition, in a recent study, loss of the G protein-coupled receptor 43 (*Gpr43*), activated specifically by AcAc in mice, abolishes IF- and KD-mediated metabolic benefits, including those associated with lipid metabolism (i.e., body weight and fat mass reduction) (Miyamoto et al., 2019). This finding suggests that the improvements seen with dietary interventions are indeed mediated at least in part by ketone body metabolism. Further studies on the ketone body-mediated epigenetic changes in dietary interventions will provide additional mechanistic understanding.

## Conclusion and Discussion of Future Perspectives

In this review, we summarized key tissue-specific epigenetic changes implicated in both metabolic diseases and dietary interventions, with a focus on adipose, liver and pancreas. While most of the epigenetic studies have been conducted separately in these metabolic tissues, it is important to recognize that the integrated tissue cross-talks can drive systemic changes in metabolic gene expression and function. For example, secretion of tissue-respective metabolites and hormones, such as adipokines (i.e., leptin, adiponectin), hepatokines (i.e., Fgf21) and pancreatic glucagon and insulin, which as we have discussed are all subject to epigenetic changes, are key mediators of tissue cross-talk and systemic homeostasis (Rosen, 2016; Stern et al., 2016). Therefore, the global metabolic changes seen in several tissues upon disease and dietary intervention make it difficult to not only characterize the adaptive or pathological role of these epigenetic events but, to also pinpoint the primary insult that triggers secondary, systemic aspects of these responses. Temporally identifying the whole-body, tissue-specific epigenetic changes throughout both disease progression and dietary intervention-mediated metabolic improvements will require further investigation. We further discuss some limitations, benefits and potentials to epigenetic modification by dietary intervention.

### Limitations of Epigenetic Modification by Dietary Intervention

#### Sex Differences

One of the key limitations of dietary interventions arises from sex differences. Despite the successful outcomes of dietary interventions seen in humans and animal models (Di Francesco et al., 2018; de Cabo and Mattson, 2019), it is not clear whether and to what extent sex differences contribute to the impact of dietary interventions. For example, in the liver, males favor energy utilization by oxidizing FA, whereas females tend to prefer energy storage by converting FA into TG (Tramunt et al., 2020). Furthermore, females primarily store excess energy in subcutaneous fat, which in comparison to visceral fat, allows for greater and longer storage, prevents ectopic fat deposition in other tissues and resists the development of male-predominant metabolic diseases, such as diabetes and NAFLD (Tramunt et al., 2020). Thus, these sex differences in metabolism could lead to different outcomes when subject to dietary interventions. Indeed, unlike male mice showing reduced lipid accumulation, IF increases the hepatic lipid content of female mice (Piotrowska et al., 2016). This result can be further explained by the sex difference in the fasting response of the liver. Upon a short-term fast (6 hr), male mice maintained steady-state metabolism with reductions in anabolic pathways, such as hepatic lipogenesis and gluconeogenesis, whereas females continued to use amino acids for the synthesis of hepatic TG (Della Torre et al., 2018). While it has been suggested that sex differences in the liver are established postnatally via testosterone-mediated DNA methylation (Reizel et al., 2015), this study with transcriptome and metabolomic analyses has demonstrated that the sexual differentiation of the liver exists when mice are born and is largely mediated by the sexually dimorphic hepatic estrogen receptor α (ERα) (Della Torre et al., 2018). Together, this evidence suggests that a comprehensive understanding of sex differences in metabolism is required for safe and efficacious utilization of fasting-involved dietary interventions in both males and females. Particularly, epigenetic changes and their regulatory roles in sexual differentiation in response to dietary interventions remain to be elucidated.

#### Age Differences

Aging is a key risk factor for metabolic disease development. Extension of lifespan and healthspan by dietary interventions has thus led to an interest in their application for the treatment of metabolic diseases in the elderly (Gensous et al., 2019). At the other extreme, pediatric obesity is also an emerging public health priority; dietary interventions are being considered for this population as well (Vidmar et al., 2019). However, as discussed in this review, most human and animal dietary intervention studies are conducted in young adult and middle-aged individuals, with very limited work in the elderly or pediatric population. Due to the stark differences in the metabolism and physiology among children, young adult and aged individuals, it is necessary to test the safety and efficacy of dietary interventions in these populations. For example, in contrast to young adult or middle-aged individuals, mildly increased body weight and/or BMI in the elderly is often associated with a lower risk of mortality. This finding, known as the “obesity paradox,” suggests a protective role of body fat in the elderly against certain illnesses (i.e., T2D) (Hainer and Aldhoon-Hainerova, 2013). In one study, weight loss (>7.5% body weight) was strongly associated with a reduced survival outcome in T2D elderly patients with a mean age of 62 years (Doehner et al., 2012). Thus, dietary interventions resulting in body weight loss could be detrimental in the elderly (Thorpe and Ferraro, 2004; Locher et al., 2016). Moreover, a study has demonstrated that 4 weeks of TRF in juvenile mice (4-week-old) causes adverse effects including delayed puberty, fatty liver disease and an abnormal gut microbiota shift (Hu et al., 2019). Therefore, the overall risk-to-benefit ratio of dietary interventions in different age populations still remains uncertain and requires further research. Moreover, it would be interesting to see how epigenetic mechanisms govern the differential response to dietary interventions among different age populations with metabolic disease.

### Benefits of Epigenetic Modification by Dietary Intervention

Despite the limitations of sex and age, the epigenetic modulation of dietary intervention is still a promising avenue. Due to the reversible nature of epigenetic modifications, the utilization of epigenetic therapies in the treatment of metabolic diseases is encouraging. Both *in vitro* and *in vivo* mechanistic studies of current epigenetic therapies, such as inhibitors of DNMT (e.g., 5-aza-2′-deoxycytidine) (Mann et al., 2007, 2010), HDAC (e.g., Trichostatin A) (Zhang et al., 2009) and HAT (e.g., Tannic acid) (Chung et al., 2019) have shown promising preclinical results in targeting hepatic disease processes. However, the low specificity and broad range of outcomes associated with epigenome-targeting agents could lead to side effects (Gius et al., 2004). Interest has also emerged in epigenetic dietary components including the DNMT/HAT inhibitor epigallocatechin-3-gallate (EGCG) found in green tea and the DNMT/HDAC inhibitor resveratrol found in peanuts, grapes and berries (Hardy and Tollefsbol, 2011). Resveratrol, commonly known as a SIRT1 activator, is used as a CR-mimetic in several studies, showing improvements in health and longevity (Baur et al., 2006). Although these natural compounds are associated with several benefits, they do not yet provide the same efficacy in achieving balanced and global effects as with dietary interventions against metabolic diseases. It will, however, be interesting to see whether combining these pharmacological or natural epigenetic modulators with dietary interventions could provide greater efficacy and reduced side effects – a perspective that can be explored in future studies. Overall, the numerous metabolic benefits associated with dietary interventions and their ability to reverse the disease state, makes them encouraging for clinical translation. Although current treatments are mainly associated with risk prevention and disease management, these dietary interventions, in combination with their epigenetic modulators, can serve to effectively improve the prognosis of metabolic diseases.

## Author Contributions

SA, NM, EM, and K-HK conceived and wrote the manuscript. SA and NM created the figures and table. All authors read and approved the final manuscript.

## Conflict of Interest

The authors declare that the research was conducted in the absence of any commercial or financial relationships that could be construed as a potential conflict of interest.
